# Natural history of Charcot-Marie-Tooth disease type 2A: a large international multicentre study

**DOI:** 10.1093/brain/awaa323

**Published:** 2021-01-08

**Authors:** Menelaos Pipis, Shawna M E Feely, James M Polke, Mariola Skorupinska, Laura Perez, Rosemary R Shy, Matilde Laura, Jasper M Morrow, Isabella Moroni, Chiara Pisciotta, Franco Taroni, Dragan Vujovic, Thomas E Lloyd, Gyula Acsadi, Sabrina W Yum, Richard A Lewis, Richard S Finkel, David N Herrmann, John W Day, Jun Li, Mario Saporta, Reza Sadjadi, David Walk, Joshua Burns, Francesco Muntoni, Sindhu Ramchandren, Rita Horvath, Nicholas E Johnson, Stephan Züchner, Davide Pareyson, Steven S Scherer, Alexander M Rossor, Michael E Shy, Mary M Reilly, for the Inherited Neuropathies Consortium - Rare Disease Clinical Research Network (INC-RDCRN), Silvia Baratta, Paula Bray, Daniela Calabrese, Kayla Cornett, Gabrielle Donlevy, Katy Eichinger, Maria Foscan, Silvia Genitrini, Natalie Rose Grant, Tara Jones, Diana Lee, Brett A McCray, Stefania Magri, Manoj Menezes, Krista Mullen, Tina Nanji, Sara Nuzzo, Emanuela Pagliano, Roy Poh, Eun Park, Saba Sadaf, Paola Saveri, Carly Siskind, Janet E Sowden, Charlotte J Sumner, Simone Thomas

**Affiliations:** 1 MRC Centre for Neuromuscular Diseases, UCL Queen Square Institute of Neurology, London, UK; 2 Department of Neurology, Carver College of Medicine, University of Iowa, Iowa City, Iowa, USA; 3 Department of Pediatric Neurosciences, Fondazione IRCCS Istituto Neurologico Carlo Besta, Milan, Italy; 4 Unit of Rare Neurodegenerative and Neurometabolic Diseases, Department of Clinical Neurosciences, Fondazione IRCCS Istituto Neurologico Carlo Besta, Milan, Italy; 5 Unit of Medical Genetics and Neurogenetics, Department of Diagnostics and Technology, Fondazione IRCCS Istituto Neurologico Carlo Besta, Milan, Italy; 6 Department of Neurology, The Perelman School of Medicine at the University of Pennsylvania, Philadelphia, PA, USA; 7 Department of Neurology, The Johns Hopkins University School of Medicine, Baltimore, Maryland, USA; 8 Connecticut Children's Medical Center, Hartford, CT, USA; 9 The Children's Hospital of Philadelphia, and Perelman School of Medicine at the University of Pennsylvania, Philadelphia, PA, USA; 10 Department of Neurology, Cedars-Sinai Medical Center, Los Angeles, CA, USA; 11 Center for Experimental Neurotherapeutics, St. Jude Children’s Research Hospital, Memphis, TN, USA; 12 Department of Neurology, University of Rochester, Rochester, NY, USA; 13 Department of Neurology, Stanford Health Care, Stanford, CA, USA; 14 Department of Neurology, Wayne State University School of Medicine, Detroit, MI, USA; 15 Department of Neurology, University of Miami Miller School of Medicine, Miami, Florida, USA; 16 Massachusetts General Hospital, Boston, Massachusetts, USA; 17 Department of Neurology, University of Minnesota, Minneapolis, Minnesota, USA; 18 University of Sydney School of Health Sciences and Children's Hospital at Westmead, Sydney, Australia; 19 Dubowitz Neuromuscular Centre, NIHR Biomedical Research Centre at UCL Great Ormond Street Institute of Child Health and Great Ormond Street Hospital, London, UK; 20 PRA Health Services, Raleigh, NC, USA; 21 Department of Clinical Neurosciences, University of Cambridge, Cambridge Biomedical Campus, Cambridge, UK; 22 Virginia Commonwealth University, Richmond, VA, USA; 23 Dr. John T. Macdonald Foundation Department of Human Genetics and John P. Hussman Institute for Human Genomics, University of Miami Miller School of Medicine, Miami, FL, USA

**Keywords:** mitofusin-2, Charcot-Marie-Tooth disease type 2A, genotype-phenotype correlations, Charcot-Marie-Tooth Examination Score v2.0, standardized response mean

## Abstract

Mitofusin-2 (MFN2) is one of two ubiquitously expressed homologous proteins in eukaryote cells, playing a critical role in mitochondrial fusion. Mutations in *MFN2* (most commonly autosomal dominant) cause Charcot-Marie-Tooth disease type 2A (CMT2A), the commonest axonal form of CMT, with significant allelic heterogeneity. Previous, moderately-sized, cross sectional genotype-phenotype studies of CMT2A have described the phenotypic spectrum of the disease, but longitudinal natural history studies are lacking. In this large multicentre prospective cohort study of 196 patients with dominant and autosomal recessive CMT2A, we present an in-depth genotype-phenotype study of the baseline characteristics of patients with CMT2A and longitudinal data (1–2 years) to describe the natural history. A childhood onset of autosomal dominant CMT2A is the most predictive marker of significant disease severity and is independent of the disease duration. When compared to adult onset autosomal dominant CMT2A, it is associated with significantly higher rates of use of ankle-foot orthoses, full-time use of wheelchair, dexterity difficulties and also has significantly higher CMT Examination Score (CMTESv2) and CMT Neuropathy Score (CMTNSv2) at initial assessment. Analysis of longitudinal data using the CMTESv2 and its Rasch-weighted counterpart, CMTESv2-R, show that over 1 year, the CMTESv2 increases significantly in autosomal dominant CMT2A (mean change 0.84 ± 2.42; two-tailed paired *t*-test *P = *0.039). Furthermore, over 2 years both the CMTESv2 (mean change 0.97 ± 1.77; two-tailed paired *t*-test *P = *0.003) and the CMTESv2-R (mean change 1.21 ± 2.52; two-tailed paired *t*-test *P = *0.009) increase significantly with respective standardized response means of 0.55 and 0.48. In the paediatric CMT2A population (autosomal dominant and autosomal recessive CMT2A grouped together), the CMT Pediatric Scale increases significantly both over 1 year (mean change 2.24 ± 3.09; two-tailed paired *t*-test *P = *0.009) and over 2 years (mean change 4.00 ± 3.79; two-tailed paired *t*-test *P = *0.031) with respective standardized response means of 0.72 and 1.06. This cross-sectional and longitudinal study of the largest CMT2A cohort reported to date provides guidance for variant interpretation, informs prognosis and also provides natural history data that will guide clinical trial design.

## Teaser

Pipis *et al*. describe the characteristics and longitudinal follow-up of 225 patients with Charcot-Marie-Tooth disease type 2A, caused by mutations in *MFN2*. They describe how different mutations affect disease onset and rate of progression and identify sensitive clinical assessments that can be used for disease monitoring.

## Introduction

Mitofusin-1 (MFN1) and mitofusin-2 (MFN2) are homologous mammalian proteins and members of the large mitochondrial transmembrane GTPase family, exhibiting ubiquitous expression in eukaryotic cells and playing a fundamental role in the dynamic mitochondrial remodelling process governed by mitochondrial fusion and fission (Chandhok *et al.*, 2018). These two highly coordinated biological processes, amongst other functions, are considered critical in mitigating mitochondrial stress, contributing to mitochondrial quality control and facilitating cellular apoptosis in cases of extreme cellular stress (Youle and van der Bliek, 2012). MFN2 is a 757-amino acid long, nuclear encoded protein (Supplementary Table 1), anchored to the outer mitochondrial membrane by two transmembrane domains (TM1 and TM2). Most of the protein, including its large dynamin-GTPase domain and two coiled-coil heptad repeat regions (HR1/cc1 and HR2/cc2), are cytosolic (Westermann, 2010; Filadi *et al.*, 2018). MFN2 is essential for mitochondrial fusion; however, the exact mechanism by which this occurs is not fully understood. A widely accepted hypothesis is that MFN2 proteins on opposing outer mitochondrial membranes mediate tethering through homologous interactions primarily of their HR2, but also HR1 regions (Filadi *et al.*, 2018). Furthermore, evidence from cultured neurons obtained from *Mfn2* knockout mice and embryonic rat and mouse neurons expressing known pathogenic *Mfn2* variants, suggest a role of MFN2 in the bidirectional axonal transport of mitochondria (Baloh *et al.*, 2007; Misko *et al.*, 2010). MFN2 also mediates sites of endoplasmic reticulum-mitochondrial contact, which are important for calcium homeostasis (de Brito and Scorrano, 2008; Merkwirth and Langer, 2008; Filadi *et al.*, 2015, 2017; Leal *et al.*, 2016).

Mutations in *MFN2* are associated with Charcot-Marie-Tooth disease type 2A (CMT2A), accounting for 4–7% of all genetically diagnosed CMT and 30–40% of genetically diagnosed axonal CMT (CMT2) (Saporta *et al.*, 2011; Murphy *et al.*, 2012; Fridman *et al.*, 2015). In comparison to CMT1A, the commonest form of CMT, CMT2A is associated with a more severe, motor-predominant phenotype that usually manifests earlier in life and carries a greater burden of disability (Feely *et al.*, 2011; Bombelli *et al.*, 2014). Additional features include optic nerve atrophy in up to 9% of patients (Chung *et al.*, 2006; Verhoeven *et al.*, 2006; Züchner *et al.*, 2006; Bombelli *et al.*, 2014), vocal cord involvement (Chung *et al.*, 2010; Kanemaru *et al.*, 2019), upper motor neuron dysfunction (Chung *et al.*, 2006; Ajroud-Driss *et al.*, 2009; Choi *et al.*, 2015; Luigetti *et al.*, 2016) and white matter lesions on MRI (Chung *et al.*, 2010; Lee *et al.*, 2017). Moreover, variants at specific amino acid positions, e.g. p.Arg104, cause a more complex syndrome with learning difficulties (Brockmann *et al.*, 2008; Del Bo *et al.*, 2008; Genari *et al.*, 2011; Tufano *et al.*, 2015; Tomaselli *et al.*, 2018).

Genotype-phenotype studies of 25–45 CMT2A cases show significant phenotypic and allelic heterogeneity (Chung *et al.*, 2006; Verhoeven *et al.*, 2006; Feely *et al.*, 2011; Bombelli *et al.*, 2014). Missense variants in the heterozygous state are frequently implicated in CMT2A and the majority of these reside within or adjacent to the dynamin-GTPase domain. GTPase domain dimerization may occur during tethering and mitochondrial fusion (Filadi *et al.*, 2018), and there is recent evidence suggesting that a dominant negative or gain-of-function pathomechanism may be responsible. *In vitro* and *in vivo* models of CMT2A have shown that certain pathogenic variants (p.Arg94Gln, p.Thr105Met) cause mitochondrial hypofusion (El Fissi *et al.*, 2018; Rocha *et al.*, 2018; Ueda and Ishihara, 2018) whilst others (p.Leu76Pro, p.Arg364Trp) cause mitochondrial hyperfusion (El Fissi *et al.*, 2018; Ueda and Ishihara, 2018). Certain variants, such as the p.Arg94Trp/Gln and p.Arg364Trp are repeatedly observed to occur *de novo* and occur in guanine and cytosine nucleotides that reside in CpG dinucleotide sequences (Chung *et al.*, 2006; Verhoeven *et al.*, 2006). Despite this, polymorphisms in *MFN2* although uncommon do occur and hence the interpretation of novel *MFN2* variants is challenging. Furthermore, autosomal recessive and semidominant cases of CMT2A have been published further illustrating the allelic heterogeneity of the condition (Nicholson *et al.*, 2008; Polke *et al.*, 2011; Tomaselli *et al.*, 2016). A recessive trait is inherited and causes disease in a recessive manner and heterozygous carriers of recessive traits are usually phenotypically normal. However, a recessive trait is considered to also be inherited in a semidominant manner when it can cause a late-onset, mild disease in the heterozygous state. Examples of variants showing semidominant inheritance in CMT2A include p.Thr362Arg (Tomaselli *et al.*, 2016) and p.Thr362Met (Nicholson *et al.*, 2008). Interestingly, the former, which is described in three further families in this study, is absent from the genome aggregation population database (gnomAD) (Karczewski *et al.*, 2020) whereas the latter is present nine times in gnomAD. Nonetheless, these are very rare variants and using the minor allele frequency in population databases to distinguish between semidominant and likely benign heterozygous variants is challenging. Interestingly, multiple symmetrical lipomatosis, a rare and phenotypically distinct disease characterized predominantly by massive increase in upper body adipose tissue and the presence of lipomata, has been associated with biallelic *MFN2* mutations (Sawyer *et al.*, 2015; Rocha *et al.*, 2017). In all cases, at least one of the *MFN2* variants is p.Arg707Trp and phenotypically some patients also manifest a late-onset axonal neuropathy.

The mitofusin knockout mouse models illustrate the biological importance of mitofusins, since strains in which either *Mfn1* or *Mfn2* are completely knocked out die *in utero* (Chen *et al.*, 2003). Furthermore, mitofusin-depleted embryonic fibroblasts show fragmented mitochondria, most likely due to the severely impaired process of mitochondrial fusion (Chen *et al.*, 2003). However, both *Mfn1* and *Mfn2* heterozygous knockout strains do not express a phenotype and demonstrate normal fertility (Chen *et al.*, 2003). There are several transgenic mouse models of CMT2A (*Mfn2^R94Q^*; *Mfn2^R94W^*; *Mfn2^T105M^*) which, nonetheless, seem to provide conflicting evidence and none of which show progressive peripheral axonal degeneration as seen in CMT2A (Detmer *et al.*, 2008; Cartoni *et al.*, 2010; Strickland *et al.*, 2014; Bannerman *et al.*, 2016; Bernard-Marissal *et al.*, 2019; Zhou *et al.*, 2019).

### Aim

This is a cross-sectional and longitudinal study of the largest CMT2A cohort reported to date which has been collected as part of the ongoing Inherited Neuropathy Consortium (INC-RDCRN) natural history study of CMT. The aim of the study is to provide genotype-phenotype correlations to aid variant interpretation, inform prognosis and to provide natural history data to guide clinical trial design.

## Materials and methods

### Ethical approvals, study design and patient recruitment

Patients included in this study were enrolled in the INC-RDCRN 6601 natural history protocol (registered at ClinicalTrials.gov NCT01193075), and 6602 and 6603 research protocols, which gained ethical approval from the institutional review boards and research ethics committees of the participating centres in the US, UK, Italy and Australia. All patients or their guardians signed the relevant consent/assent forms. Patients were evaluated at one of the 19 INC centres between 2009 and 2019 and at Wayne State University between 1996 and 2009. Antecedent clinical data were collected retrospectively from the patient history. Longitudinal follow-up data (clinical history and examination with or without neurophysiological studies) was collected prospectively during annual visits.

### *MFN2* variant curation and classification

For conciseness, we hereafter use the term autosomal dominant (AD) CMT2A (AD-CMT2A) to refer to all the cases that carry a heterozygous variant in *MFN2*, irrespective of whether the variant was parentally inherited or occurred *de novo*. In our study, we used pathogenicity criteria from both pathogenic and benign categories (Supplementary Table 2) as published in the American College of Medical Genetics and Genomics and Association for Molecular Pathology (ACMG/AMP) guidelines (Richards *et al.*, 2015), and classified all *MFN2* variants into pathogenic, likely pathogenic and variants of uncertain significance (Supplementary Tables 3 and 4). The most recent Association for Clinical Genomic Science (ACGS) recommendations on variant classification (Ellard *et al.*, 2020) were also taken into consideration to appropriately use downgraded or upgraded pathogenicity criteria in cases/pedigrees that could not be fully classified on the ACMG/AMP guidance alone. The ACMG/AMP guidelines suggest using the PP1 criterion (segregation data) as a stronger evidence with increasing segregation data; however, they do not provide a quantification for this. Therefore, we used the Jarvik and Browning guidance (Jarvik and Browning, 2016) on how to quantify segregation data from multiple affected family members and appropriately assign pathogenicity criteria to that variant. Briefly, the product of all informative meioses across all affected members from all unrelated families was used to determine if the segregation criterion can be used as a supporting (PP1), moderate (PP1_moderate) or strong (PP1_strong) level of evidence based on probabilistic preset cut-offs between the three categories. We also used the REVEL meta-predictor tool, which combines pathogenicity and conservation scores from individual *in silico* prediction tools (Ioannidis *et al.*, 2016). The REVEL-derived aggregate score is more accurate compared to the combination of individual tools, which often assess overlapping subsets of evidence, thus inadvertently leading to ‘double-counting’ of evidence.

The presence of a variant in the gnomAD population database (Karczewski *et al.*, 2020) was considered in the context of the disease prevalence, penetrance, genetic and allelic heterogeneity of CMT. A threshold of an allele count of 3 in gnomAD was used to distinguish between heterozygous variants that are plausible or not to be causal for CMT2A (Pipis *et al.*, 2019). Furthermore, all the variants described in autosomal recessive CMT2A cases (AR-CMT2A) in this study (Supplementary Table 5) had a gnomAD population allele frequency that would be compatible with the genetic architecture of autosomal recessive CMT as previously published (Pipis *et al.*, 2019). We have also detailed the benign (B) and likely benign (LB) variants we have encountered in our study and the reasons for their classifications (Supplementary Table 6).

Previously published case series and case reports of CMT2A were identified through an extensive PubMed literature review, the Inherited Neuropathy Variant Browser (Saghira *et al.*, 2018) and ClinVar (Landrum *et al.*, 2014).

### CMT clinical outcome measures

The clinical outcome measures used in this study included the CMT Neuropathy Score version 2 (CMTNSv2) and the Rasch modified CMTNSv2 (CMTNSv2-R), both composite scores based on patients’ symptoms (three items), examination findings (four items), and electrophysiology (two items) (Murphy *et al.*, 2011; Sadjadi *et al.*, 2014). The CMT Examination Score version 2 (CMTESv2) and Rasch modified version (CMTESv2-R) are subscores of the CMTNSv2 comprising seven items from the patients’ symptoms and examination findings. The psychometrics of the Rasch weighting have only been performed in CMT1A patients (Sadjadi *et al.*, 2014) and hence we used CMTESv2 as our primary clinical outcome measure for this study. Most assessment visits did not include neurophysiological studies and only the CMTESv2 and CMTESv2-R were obtained during these visits. Therefore, to maximize the sample size during the statistical analysis of the longitudinal data, the CMTESv2 and CMTESv2-R were primarily analysed; a similar analysis approach was also used in a CMT1A natural history study (Fridman *et al.*, 2020). We also analysed data obtained from the CMT Pediatric Scale (CMTPedS), a well-validated composite score that assesses strength, hand dexterity, sensation, gait, balance, power and endurance in children with CMT from the age of 3 years (Burns *et al.*, 2012). For each of these scales a higher score indicates a higher level of impairment. Clinical investigators at each site received training in the administration of the clinical outcome measures and were certified clinical investigators prior to use.

### Statistical analysis

Data were analysed mostly on an available basis. The chi-squared goodness-of-fit test (skewness, kurtosis, median, mean) was used to ascertain the distribution of data. The baseline demographics and characteristics, clinical data from the history and examination and the physical disability were analysed using descriptive statistics. Throughout the manuscript values describing continuous data represent the mean ± standard deviation (SD). Correlations between categorical data at baseline were assessed with chi-squared (χ^2^) or Fisher’s exact test as appropriate. Correlations between the CMTESv2 and the disease duration as calculated at the baseline visit were assessed with two-tailed, Spearman’s rank correlation coefficient. The longitudinal responsiveness of the CMTESv2, CMTESv2-R and CMTPedS was quantified as the standardized response mean [SRM = mean change/standard deviation (SD) change]. SRM-values of 0.20–0.49, 0.50–0.79, and ≥0.80 correspond to small, moderate, and large responsiveness, respectively as suggested originally (Cohen, 1988). A two-tailed, paired Student’s *t*-test was used to compare longitudinal changes in the CMTESv2, CMTESv2-R and CMTPedS. *P *≤* *0.05 was considered statistically significant.

### Data availability

The data that support the findings of this study are available from the corresponding author, upon reasonable request. The data are not publicly available since they contain information that could compromise the privacy of research participants.

## Results

A total of 225 patients with *MFN2* variants were recruited in 19 INC centres in the US, UK, Italy and Australia (100 males: 125 females). Eighty-seven of these were children under the age of 20 years (43 males: 44 females) and the average age ± SD at enrolment for the entire cohort was 31.30 ± 19.88 years. The age distribution was considered as a single mode with an arbitrary cut-off at the age of 20 years, rather than bimodal, as there are no distinct paediatric and adult presentations in CMT2A.

We identified 179 patients from 133 families with dominant pathogenic (ACMG class 5) or likely pathogenic (class 4) *MFN2* variants (AD-CMT2A; Supplementary Table 3), and 17 patients from 13 families with AR-CMT2A (Supplementary Table 5); 13 of 17 patients with AR-CMT2A harboured homozygous variants or compound heterozygous variants proven to be in *trans* phase. Twenty-nine patients from 23 families with variants of uncertain significance are also reported (Supplementary Table 4). For both the cross-sectional genotype-phenotype and longitudinal studies, only cases with pathogenic or likely pathogenic variants were considered.

### Disease presentation and variant topology

**Figure 1:**
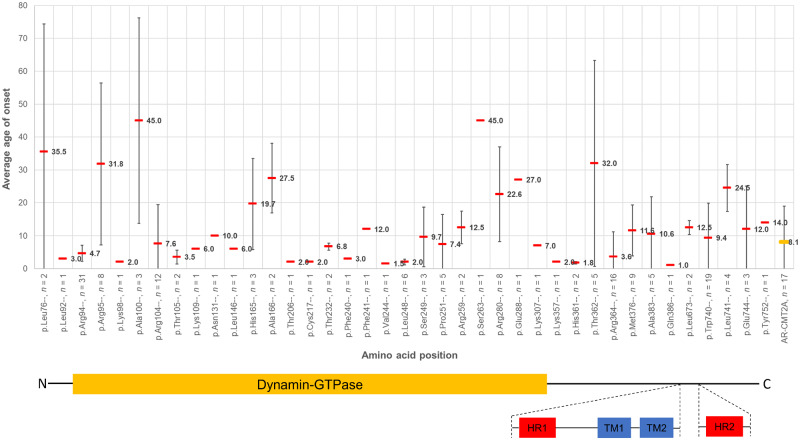
**The average age of onset of symptoms at each amino acid position for all the pathogenic and likely pathogenic variants with dominant inheritance and AR-CMT2A cases identified in this study.** AR-CMT2A cases shown on the *far right*. For each amino acid position, the cumulative variant count irrespective of the amino acid substitution, is also listed (*n*; for example ‘p.Arg94–, *n = *31’ contains all 31 cases of p.Arg94Trp, p.Arg94Gln, p.Arg94Gly and p.Arg94Leu). On the *x*-axis, the variants are equidistant from one another for graphical purposes; the distance between the variants displayed is not to scale. However, the primary structure of the MFN2 protein has been drawn below the graph in a skewed fashion to account for this and highlights which functional domains the variants reside in. The average age of onset of symptoms for each amino acid position is displayed with a horizontal red bar and the standard deviation bars, indicative of the spread in age of onset for each position, are also shown for all amino acid positions with variant counts of two or more.

Most patients with AD-CMT2A and AR-CMT2A first noticed symptoms in the first two decades of life, usually walking or balance difficulties. For both modes of inheritance, the correlation of the average age of onset with the genotype (amino acid position) is illustrated in Fig. 1 with variants at most amino acid positions being associated with symptom onset at or before the age of 15 years. Many of these variants reside in the dynamin-GTPase domain even after standardizing for the size of the domain. Although most of these are associated with early onset disease, there is considerable phenotypic heterogeneity within the domain with respect to the age of onset even for variants in adjacent positions. Furthermore, variants at specific amino acid positions that are usually associated with early onset disease (p.Arg94, p.Arg104, p.Leu248, p.Arg364, p.Trp740) also seem to be associated with a tight time window during which the disease manifests. Patients with AR-CMT2A almost always had disease onset and first symptoms in childhood with an average age of onset of 8.06 years ± 10.92 years (SD); one exceptional case stands out who despite careful questioning could not time the onset of his symptoms before his late 40s. His brother who also carries the same variants in *trans* suffers from early onset AR-CMT2A and both suffer from a moderate burden of disease in their sixth decade of life (AR8 pedigree in Supplementary Table 5; Tomaselli *et al.*, 2016).

In this cohort, and in line with previous publications, mutations in certain amino acid positions are always pathogenic, with no evidence of reduced penetrance or variable expressivity (range of phenotypic expression) despite different amino acid substitutions. Examples of different missense changes being observed at the same conserved amino acid position in our cohort include p.Arg94Trp/Gln, p.Arg104Glu/Trp, p.Ser249Thr/Cys and p.Trp740Ser/Arg. On the contrary, mutations in other amino acid positions are associated with phenotypic heterogeneity (early versus later onset of disease) dependent on the amino acid substitution at the same position. An example from our cohort is the group of 13 patients carrying the known p.Arg364Trp variant which is associated with early onset and severe disease and a single proband carrying the p.Arg364Gln variant who first presented with symptoms at the age of 32 years and had a recorded CMTESv2 of 8 at the age of 55 years, consistent with mild disease.

### Genotype-phenotype study

The clinical characteristics of patients (Tables 1 and 2) were captured at their baseline visit and were analysed after classifying by inheritance pattern, age of onset, variant topology and the biological effect of variants. Inheritance pattern was defined as AD-CMT2A versus AR-CMT2A, and age of onset was defined as childhood onset if disease presentation occurred between 1 and 20 years versus adult onset if the presentation occurred after 21 years (analysed in AD-CMT2A patients). Variant topology was defined as variants within the dynamin-GTPase domain versus those outside the domain and the biological effect of variants was defined as those variants shown to cause mitochondrial hypofusion in non-human disease models (p.Arg94Gln, p.Thr105Met) versus variants shown to cause mitochondrial hyperfusion (p.Leu76Pro, p.Arg364Trp).

**Table 1:** Baseline clinical characteristics classified by inheritance pattern and age of onset in 179 patients with pathogenic and likely pathogenic dominant variants (AD-CMT2A) and 17 patients with AR-CMT2A

Clinical characteristics	Inheritance pattern		Onset in AD-CMT2A	
	AD-CMT2A	AR-CMT2A	Childhood onset (1–20 years)	Adult onset (>20 years)
Patients, *n*	179	17	144	26
Age of symptom onset, years, mean ± SD	11.61 ± 15.07 (range 1.0–81)	8.06 ± 10.92 (range 1.5–48)	**6.15 ± 4.82 (range 1–20)**	**41.88 ± 16.63 (range 23–81)**
Disease duration, years, mean ± SD	19.98 ± 14.84 (range 0.1–65.2)	25.35 ± 12.36 (range 4.3–46.1)	20.86 ± 15.56 (range 0.1–65.2)	15.15 ± 8.67 (range 0.7–33.3)
Delayed walking (> 15 months), *n*	22/148 (15%)	3/10 (30%)	18/126 (14%)	2/15 (13%)
Foot deformities, *n*	**127/160 (79%)**	**8/15 (53%)**	102/128 (80%)	19/25 (76%)
Ankle-foot orthoses, *n*	**104/166 (63%)**	**14/15 (93%)**	**91/133 (68%)**	**10/25 (40%)**
Walking aids, *n*	46/161 (29%)	7/15 (47%)	38/130 (29%)	8/23 (35%)
Wheelchair-dependent, *n*	43/163 (26%)	3/15 (20%)	**41/132 (31%)**	**1/23 (4%)**
Foot surgery, *n*	**42/166 (25%)**	**8/15 (53%)**	39/134 (29%)	3/25 (12%)
Dexterity difficulties, *n*	106/166 (64%)	11/14 (79%)	**92/132 (70%)**	**12/26 (46%)**
Optic nerve atrophy, *n*	12/168 (7%)	3/15 (20%)	12/134 (9%)	0/25 (0%)
Hearing loss, *n*	11/159 (7%)	1/14 (7%)	8/126 (6%)	3/25 (12%)
Scoliosis, *n*	19/162 (12%)	4/14 (29%)	16/131 (12%)	3/24 (13%)
CMTESv2	**10.75 ± 6.90 (157)**	**14.57 ± 6.07 (14)**	**12.06 ± 6.82 (122)**	**6.50 ± 3.42 (26)**
CMTESv2-R	**14.27 ± 8.05 (157)**	**19.14 ± 6.56 (14)**	**15.82 ± 7.71 (122)**	**9.42 ± 5.15 (26)**
CMTNSv2	15.27 ± 9.71 (90)	21.00 ± 5.20 (7)	**17.20 ± 9.79 (71)**	**8.47 ± 4.93 (15)**
CMTNSv2-R	19.13 ± 10.73 (90)	26.14 ± 5.61 (7)	**21.42 ± 10.47 (71)**	**11.13 ± 6.45 (15)**
CMTPedS	26.45 ± 10.26 (47)	27.00 ± 11.31 (2)	27.31 ± 9.57, (45)	n/a

Of 179 patients with pathogenic and likely pathogenic dominant variants, five were asymptomatic at the age of assessment and four patients had an unknown age of onset of symptoms. Continuous and categorical data that are highlighted in bold in the inheritance pattern and AD-CMT2A groups, indicate a statistically significant difference (*P *<* *0.05) between the observed values within those groups. The CMTPedS was only performed in patients aged ≤20 years at the time of assessment. Data in the bottom five rows showing the clinical outcome scores represent mean ± SD (*n*). All percentage values are rounded to the nearest whole point. n/a = not applicable.

**Table 2:** Baseline clinical characteristics classified by variant topology and biological effect of the variant in patients with pathogenic and likely pathogenic dominant variants (AD-CMT2A)

Clinical characteristics	Variant topology (AD-CMT2A)		Biological effect of variants	
	GTPase domain variants	Non-GTPase domain variants	Mitochondrial hypofusion (R94Q, T105M)	Mitochondrial hyperfusion (L76P, R364W)
Patients, *n*	104	75	11	17
Age of symptom onset, years, mean ± SD	11.79 ± 15.35 (range 1–81)	11.37 ± 14.76 (range 1–70)	5.55 ± 2.97 (range 2.5–12)	5.71 ± 14.85 (range 1–63)
Disease duration, years, mean ± SD	19.03 ± 13.99 (range 0.1–53.1)	21.31 ± 15.96 (range 2.6–65.2)	19.19 ± 12.67 (range 3.6–43.7)	17.51 ± 16.68 (range 2.7–49.3)
Foot deformities, *n*	73/93 (78%)	54/67 (81%)	7/8 (88%)	11/14 (79%)
Ankle-foot orthoses, *n*	**67/96 (70%)**	**37/70 (53%)**	9/10 (90%)	14/16 (88%)
Walking aids, *n*	24/93 (26%)	22/68 (32%)	4/10 (40%)	8/16 (50%)
Wheelchair-dependent, *n*	25/96 (26%)	18/67 (27%)	4/10 (40%)	9/16 (56%)
Foot surgery, *n*	29/95 (31%)	13/71 (18%)	5/10 (50%)	2/17 (12%)
Dexterity difficulties, *n*	**68/97 (70%)**	**38/69 (55%)**	8/10 (80%)	11/15 (73%)
Optic nerve atrophy, *n*	10/99 (10%)	2/69 (3%)	0/11 (0%)	2/14 (14%)
Hearing loss, *n*	3/90 (3%)	8/69 (12%)	0/10 (0%)	2/13 (15%)
Scoliosis, *n*	**15/94 (16%)**	**4/68 (6%)**	0/9 (0%)	3/16 (19%)
CMTESv2	11.31 ± 7.06 (87)	10.06 ± 6.68 (70)	12.13 ± 7.02 (8)	16.73 ± 7.81 (15)
CMTESv2-R	15.15 ± 8.16 (87)	13.19 ± 7.82 (70)	16.25 ± 8.00 (8)	20.07 ± 8.46 (15)
CMTNSv2	16.68 ± 10.38 (47)	13.72 ± 8.79 (43)	20.50 ± 16.26 (2)	26.00 ± 8.26 (10)
CMTNSv2-R	20.98 ± 11.30 (47)	17.12 ± 9.81 (43)	25.00 ± 16.97 (2)	29.90 ± 8.20 (10)
CMTPedS	27.88 ± 8.17 (24)	24.96 ± 12.06 (23)	27.00 ± 7.07 (2)	34.78 ± 5.97 (9)

The amino acid positions used for the dynamin-GTPase domain are 93–342. Variants shown to cause mitochondrial hypofusion in non-human disease models are p.Arg94Gln (R94Q) and p.Thr105Met (T105M), whereas variants shown to cause mitochondrial hyperfusion are p.Leu76Pro (L76P) and p.Arg364Trp (R364W). Categorical data highlighted in bold in the variant topology group indicate a statistically significant difference (*P *<* *0.05) between the observed values within that group. The CMTPedS was only performed in patients aged ≤20 years at the time of assessment. Data in the bottom five rows showing the clinical outcome scores represent mean ± SD (*n*). All percentage values are rounded to the nearest whole point.

Comparing AD-CMT2A to AR-CMT2A (Table 1), both groups had a similar age of onset of symptoms [average 11.61 years ± 15.07 years (SD) versus average 8.06 years ± 10.92 years (SD), two-tailed Mann-Whitney U-value 1291.5, *P = *0.472] and at the baseline assessment had no significant difference in their disease duration [average 19.98 years ± 14.84 years (SD) versus average 25.35 years ± 12.36 years (SD), two-tailed Mann-Whitney U-value 1057.5, *P = *0.069]. Disease duration was calculated from the age at the baseline visit minus the age of onset of symptoms. A minority of patients in both groups had delayed walking milestones (walked after the 15th month) but there was no significant difference between this percentage in the two groups (15% versus 30%, Fisher’s exact test *P = *0.196). Despite a similar disease duration between the two groups, a significantly higher proportion of patients with AD-CMT2A had foot deformities (79% versus 53%, Fisher’s exact test *P = *0.047), but a significantly higher proportion of patients with AR-CMT2A were using ankle-foot orthoses (63% versus 93%, χ^2^ test *P = *0.017) and had undergone foot surgery (25% versus 53%, Fisher’s exact test *P = *0.032). Overall, patients with AR-CMT2A had a significantly higher mean CMTESv2 at baseline [10.75 ± 6.90 (SD) versus 14.57 ± 6.07 (SD), two-tailed Mann-Whitney U-value 708.5, *P = *0.028], as well as a higher mean CMTESv2-R indicating a higher burden of accrued disability over the same time-frame. Both groups had a moderate mean CMTPedS score [AD-CMT2A mean 26.45 ± 10.26 (SD) and AR-CMT2A mean 27.00 ± 11.31 (SD)] but with no significant difference between them. There were no statistically significant differences between the two groups in the use of walking aids (29% versus 47%) or wheelchair (26% versus 20%), dexterity difficulties (64% versus 79%), optic nerve atrophy (7% versus 20%), hearing loss (both 7%) or scoliosis (12% versus 29%).

Of 179 patients with pathogenic or likely pathogenic autosomal dominant variants (AD-CMT2A), 170 had available data on age of onset and the majority had onset in childhood (*n = *144; Table 1). In both groups of patients, a minority had delayed walking milestones but there was no significant difference in this percentage between the two groups (14% versus 13%, Fisher’s exact test *P = *1). Despite similar disease duration between childhood and adult-onset AD-CMT2A at the baseline visit [average 20.86 years ± 15.56 years (SD) versus average 15.15 years ± 8.67 years (SD); two-tailed Mann-Whitney U-value 1592.5, *P = *0.226], there was a significantly higher percentage of patients with childhood onset AD-CMT2A compared to adult onset AD-CMT2A, using ankle-foot orthoses (68% versus 40%, χ^2^ test *P = *0.007), wheelchair-dependent (31% versus 4%, χ^2^ test *P = *0.008) and with impaired dexterity (defined as either difficulties with cutlery or difficulty with buttons or both; 70% versus 46%, χ^2^ test *P = *0.021). Furthermore, patients with childhood-onset AD-CMT2A had a significantly higher mean of CMTESv2 [12.06 ± 6.82 (SD) versus 6.50 ± 3.42 (SD), two-tailed Mann-Whitney U-value 787, *P < *0.001], CMTESv2-R, CMTNSv2 [17.20 ± 9.79 (SD) versus 8.47 ± 4.93 (SD), two-tailed Mann-Whitney U-value 249.5, *P = *0.001] and CMTNSv2-R over a similar disease duration compared to adult-onset AD-CMT2A. Patients with childhood onset AD-CMT2A had a moderate mean CMTPedS score of 27.31 ± 9.57 (SD) at baseline. There was no significant difference in the prevalence of foot deformities between the two groups (80% versus 76%), the use of walking aids (29% versus 35%), the prevalence of previous foot surgery (29% versus 12%), hearing loss (6% versus 12%) or scoliosis (12% versus 13%).

In an analysis of AD-CMT2A cases with variants residing within the dynamin-GTPase domain (amino acid positions 93–342) versus AD-CMT2A cases with variants outside the domain (Table 2), there were no significant differences in the age of onset of symptoms, baseline mean CMTESv2, CMTESv2-R, CMTNSv2, CMTNSv2-R and CMTPedS scores and both groups had similar disease duration periods. Nonetheless, a slightly higher proportion of patients with variants in the dynamin-GTPase domain used ankle-foot orthoses (70% versus 53%, χ^2^ test *P = *0.026), complained of dexterity difficulties (70% versus 55%, χ^2^ test *P = *0.047) and developed scoliosis (16% versus 6%, χ^2^ test *P = *0.049).

Analysis of patients harbouring variants that have been shown to cause mitochondrial hypofusion (p.Arg94Gln, p.Thr105Met) or hyperfusion (p.Leu76Pro, p.Arg364Trp), showed no significant difference in the age of onset, disease duration, mean CMTESv2, CMTESv2-R, CMTNSv2, CMTNSv2-R and CMTPedS scores (Table 2).

In our cohort, optic nerve atrophy was reported in a total of 15 patients: 12 patients with AD-CMT2A (of a total of 168 who had documented optic nerve assessment, 7.1%) and three patients with AR-CMT2A (of a total of 15 who had documented optic nerve assessment, 20%). Despite the higher percentage in the AR-CMT2A group, this was not statistically significant (Fisher’s exact test *P = *0.111). Furthermore, in patients with AD-CMT2A, optic atrophy was most frequently involved with variants at the amino acid position p.Arg104 (*n = *4), followed by p.Arg364Trp (*n = *2) and p.Leu248 (*n = *2). None of the category groups of age of onset of symptoms (child versus adult onset AD-CMT2A), variant topology (GTPase domain variants versus non-GTPase domain variants) or the biological effect of certain variants on mitochondrial fusion dynamics (hypofusion versus hyperfusion variants) had statistically significant enrichment with cases of CMT2A and concurrent optic atrophy.

### Disease progression

**Figure 2:**
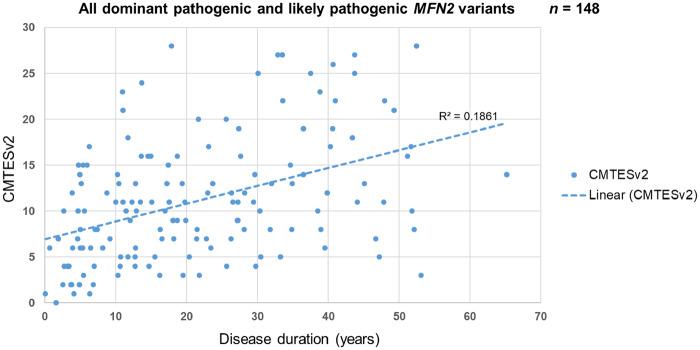
**Correlation of CMTESv2 and disease duration as surrogate evidence of disease progression.** The disease duration was calculated by subtracting the age of onset from the age at assessment at the baseline visit. The dashed line represents the linear regression coefficient (R^2^). 148 patients with a dominant pathogenic or likely pathogenic *MFN2* variant had CMTESv2 data at their baseline visit; the correlation between CMTESv2 and disease duration is statistically significant (two-tailed Spearman’s ρ 0.44, *P < *0.001).

CMT2A is a progressive disease with regards to length-dependent weakness and sensory loss and a cross-sectional analysis of the baseline data of patients with AD-CMT2A shows a statistically significant correlation between disease duration and the CMTESv2 (Fig. 2); this illustrates a worsening CMTESv2 as the disease progresses (two-tailed Spearman’s ρ = 0.44, *P < *0.001). Correlation of the CMTESv2-R with disease duration showed similar results with a parallel linear regression coefficient line (not shown). It is important to also note that this correlation includes patients with variants that are known to be associated with early and late onset disease, and with varying degrees of pace of progression.

**Figure 3:**
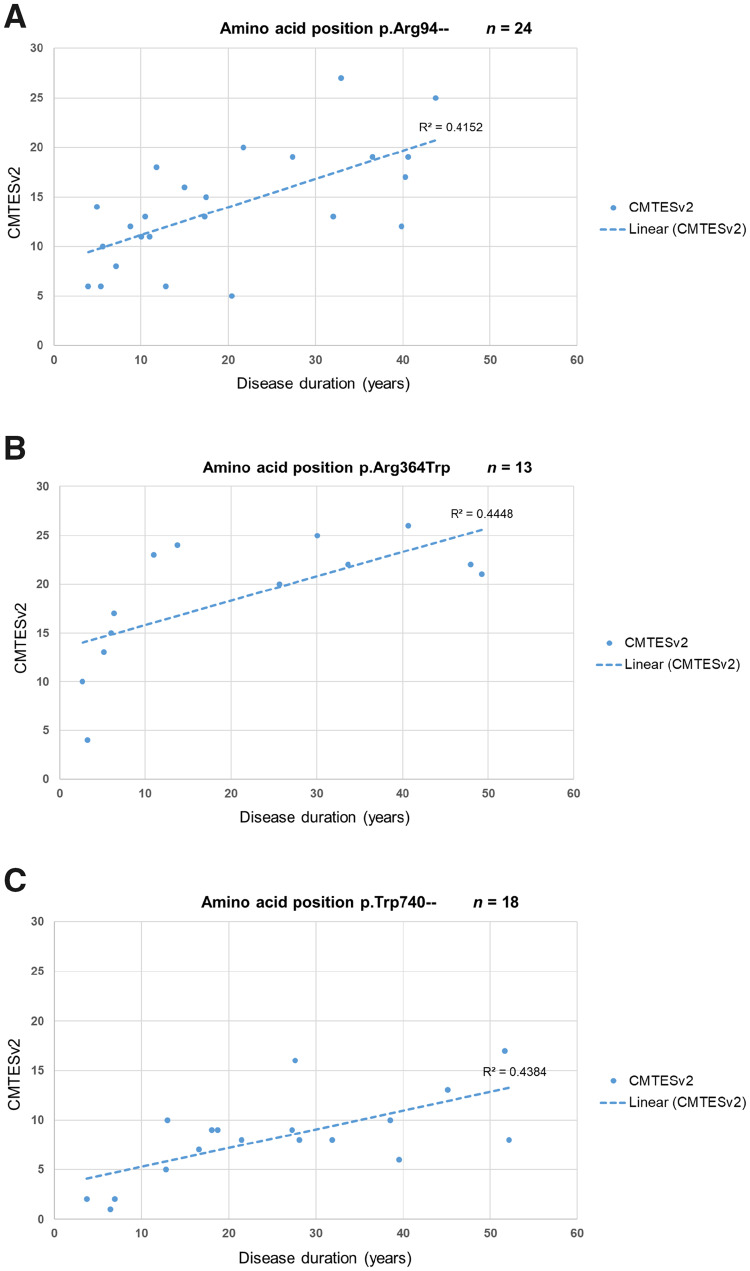
**Correlation of CMTESv2 and disease duration in the three commonest missense variants causing CMT2A.** The disease duration was calculated by subtracting the age of onset from the age at assessment at the baseline visit. The dashed line represents the linear regression coefficient (R^2^). (**A**) 24 patients with a heterozygous mutation at the p.Arg94– amino acid position had CMTESv2 data at their baseline visit. This subgroup includes patients with the variants p.Arg94Trp, p.Arg94Gln, p.Arg94Gly and p.Arg94Leu; the correlation between CMTESv2 and disease duration in this group is statistically significant (two-tailed Spearman’s ρ 0.65, *P < *0.001). (**B**) Thirteen patients carrying the heterozygous variant p.Arg364Trp had CMTESv2 data at their baseline visit and the correlation between CMTESv2 and disease duration is statistically significant (two-tailed Spearman’s ρ 0.72, *P = *0.0054). (**C**) Eighteen patients with a heterozygous variant at the p.Trp740– amino acid position had CMTESv2 data at their baseline visit and the correlation between CMTESv2 and disease duration is statistically significant (two-tailed Spearman’s ρ 0.58, *P = *0.011).

**Figure 4:**
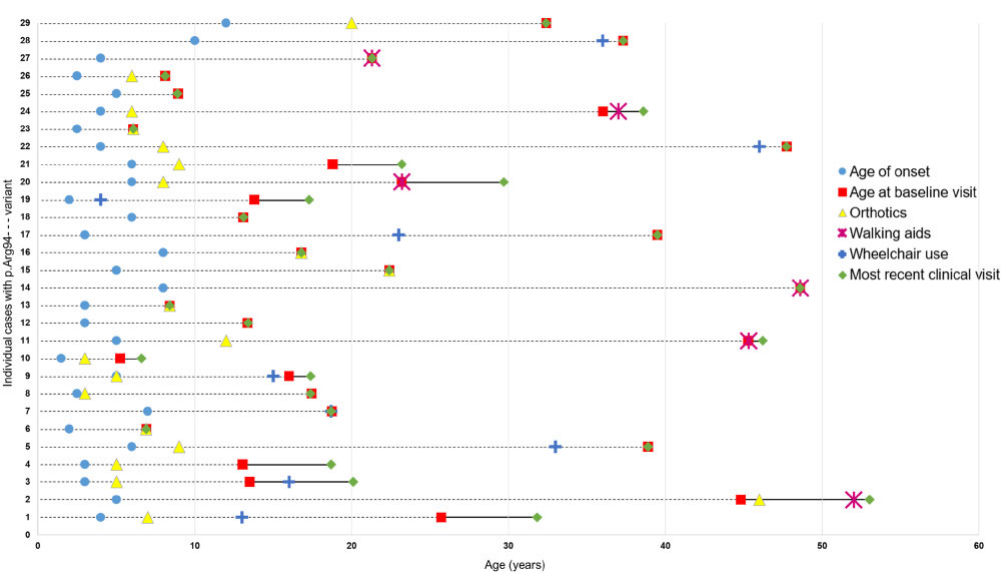
**Genotype-physical impact correlation in 29 patients with CMT2A due to pathogenic and likely pathogenic variants at the p.Arg94– amino acid position.** Each horizontal set of data-points plotted over time (age in years) corresponds to one of the 29 patients (1–29 on the *y*-axis). Patients 1–16 carry the p.Arg94Trp variant, Patient 17 carries the p.Arg94Leu variant, Patients 18 and 19 carry the p.Arg94Gly variant, and Patients 20–29 carry the p.Arg94Gln variant. In each set of data-points, the red square represents the age at which the patient was first seen and enrolled in the study (baseline visit): the dashed line to its *left* represents retrospective information gathered by history and the solid line to its *right* represents prospective information gathered during the entirety of the study. The green diamond represents the last age with available clinical data. Most of the patients have only been seen once and hence the red square and green diamond coincide. The blue circle represents the age of onset of symptoms in the patient, the yellow triangle represents the age at which ankle-foot orthoses were needed and prescribed, the purple star represents the age at which walking aids (stick, walker) were required and the blue cross represents the age at which the patient reverted to the regular use of a wheelchair.

According to the current study and previously published studies, the amino acid positions p.Arg94, p.Arg364 and p.Trp740 are the three commonest residues for the occurrence of missense variants in *MFN2* causing CMT2A. Patients with variants in the amino acid position p.Arg94, which is the most common of the three, show a significant correlation of baseline CMTESv2 with disease duration (two-tailed Spearman’s ρ  =  0.65, *P < *0.001) (Fig. 3A). A detailed assessment of the disease impact over time in these 29 patients with regards to the use of ankle-foot orthoses, walking aids and wheelchair use is presented in Fig. 4. Almost all patients require ankle-foot orthoses in the first two decades of life, with most prescriptions given in childhood and of the seven patients requiring regular use of a wheelchair, this was before the age of 40 years in six. Patients carrying the p.Arg364Trp variant (Fig. 3B) also show a significant correlation between baseline CMTESv2 and disease duration (two-tailed Spearman’s ρ 0.72, *P = *0.005). However, these patients have more severe disease early on and throughout the entirety of the disease compared to p.Arg94 as illustrated by the higher CMTESv2 scores and have a slightly faster pace of progression. On the contrary, patients with variants at the p.Trp740 position (Fig. 3C) have milder disease early on and throughout the entirety of the disease compared to p.Arg94 and p.Arg364 as illustrated by the lower CMTESv2 scores. Patients with variants at the p.Trp740 position also show a significant correlation between baseline CMTESv2 and disease duration (two-tailed Spearman’s ρ 0.58, *P = *0.011).

Of the 179 patients with AD-CMT2A, 92 patients had longitudinal data, of whom 38 had 1-year follow-up data and 34 had 2-year follow-up data. Eight patients with AR-CMT2A had longitudinal data, of whom six had 1-year follow-up data, four had 2-year follow-up data and five had 4-year follow-up data. The longitudinal data from the CMTESv2, the weighted CMTESv2-R and the CMTPedS of all AD-CMT2A and AR-CMT2A patients was used to calculate the mean change over 1 and 2 years for each group respectively. Subsequently the aggregated data were used to calculate the SRM of the CMTESv2, CMTESv2-R and CMTPedS over 1 and 2 years (Table 3). The SRM is a metric that describes how sensitive a particular outcome is to change. In patients with AD-CMT2A, the CMTESv2 increased significantly over 1 year [mean change 0.84 ± 2.42 (SD), two-tailed paired *t*-test *P = *0.039]; the equivalent 1-year SRM at 0.35 showed small responsiveness. Surprisingly, the CMTESv2-R did not show a significant change over 1 year [mean change 0.63 ± 3.19 (SD), two-tailed paired *t*-test *P = *0.230]. Over 2 years, the CMTESv2 increased significantly [mean change 0.97 ± 1.77 (SD), two-tailed paired *t*-test *P = *0.003], as did the CMTESv2-R [mean change 1.21 ± 2.52 (SD), two-tailed paired *t*-test *P = *0.009], and their 2-year SRM values of 0.55 and 0.48 reflect moderate and small responsiveness, respectively. Analysis of the CMTPedS in all the paediatric AD-CMT2A and AR-CMT2A cases grouped together, showed that it increased significantly over 1 year (mean change 2.24 ± 3.09; two-tailed paired *t*-test *P = *0.009) and over 2 years (mean change 4.00 ± 3.79; two-tailed paired *t*-test *P = *0.031) with respective SRMs of 0.72 and 1.06. There was no significant change in the CMTESv2 or CMTESv2-R in the AR-CMT2A group and this is likely to be due to the small sample size of patients with available follow-up data from baseline.

**Table 3:** Mean change and SRM of CMTESv2, CMTESv2-R and CMTPedS in AD-CMT2A and AR-CMT2A at 1- and 2-years follow-up

	Follow-up, years	*n*	Baseline	Change	*P*	SRM
Autosomal dominant						
CMTESv2	1	38	10.66 ± 5.94	0.84 ± 2.42	**0.039**	**0.35**
CMTESv2R	1	38	14.53 ± 6.96	0.63 ± 3.19	0.230	0.20
CMTESv2	2	34	9.47 ± 6.26	0.97 ± 1.77	**0.003**	**0.55**
CMTESv2-R	2	34	13.00 ± 7.68	1.21 ± 2.52	**0.009**	**0.48**
Autosomal recessive						
CMTESv2	1	6	12.33 ± 6.65	0.17 ± 3.06	0.900	0.05
CMTESv2-R	1	6	17.00 ± 7.21	−0.33 ± 2.73	0.777	−0.12
CMTESv2	2	4	10.25 ± 7.23	0.25 ± 1.26	0.718	0.20
CMTESv2-R	2	4	14.75 ± 7.63	0 ± 1.41	1.000	0
CMTESv2	4	5	13.40 ± 6.66	1.80 ± 3.11	0.266	0.58
CMTESv2R	4	5	17.60 ± 7.23	1.40 ± 3.91	0.468	0.36
Autosomal dominant and autosomal recessive						
CMTPedS	1	17	25.41 ± 8.01	2.24 ± 3.09	**0.009**	**0.72**
CMTPedS	2	7	28.86 ± 7.82	4.00 ± 3.79	**0.031**	**1.06**

The CMTESv2 changed significantly over 1 and 2 years in patients with AD-CMT2A, whereas the CMTESv2-R changed significantly over 2 years. The CMTPedS (all cases grouped) changed significantly both over 1 and 2 years. There was no significant change in cases with AR-CMT2A over 1 and 2 years. Mean changes that are statistically significance have their *P*-values and SRMs highlighted in bold. Data shown represent mean ± SD.

## Discussion

In this large international prospective study of 196 patients with CMT2A, the majority of pathogenic and likely pathogenic variants occur in the dynamin-GTPase domain of MFN2, which plays a central role in mitochondrial fusion (Westermann, 2010; Chandhok *et al.*, 2018). In the gnomAD population database (Karczewski *et al.*, 2020), of the 30 most frequent missense variants observed in *MFN2*, each with an allele count (AC) of >9 (range AC 9–1942), only three are situated in the dynamin-GTPase domain (p.Gly298Arg AC = 606, p.Cys281Ser AC = 92, p.Arg250Gln AC = 62).

There are earlier reports associating nonsense *MFN2* variants (E65X, R418X) in the heterozygous state with CMT2A and since such truncated transcripts are expected to undergo nonsense mediated decay, this would suggest that MFN2 may be intolerant of haploinsufficiency. However, evidence from the *Mfn2* heterozygous knockout mice that do not express a phenotype (Chen *et al.*, 2003) and more recent evidence from human models of the disease would argue against this. The mother of a proband with AR-CMT2A, who is a heterozygous carrier of the p.Glu308X variant and is unaffected by history and neurophysiology (EMG) at the age of 39, had RT-PCR transcript analysis from blood which indicated that the truncated transcript undergoes nonsense-mediated decay (NMD) (Polke *et al.*, 2011). The lack of a phenotype in her (at least until mid-adulthood) would suggest that MFN2 tolerates haploinsufficiency. An exception to this are nonsense *MFN2* variants in the last exon, which are expected and have been shown to escape NMD (Kawarai *et al.*, 2016) and in line with this, heterozygous carriers of the variants p.751X and p.752X develop an early onset severe CMT2A phenotype (Verhoeven *et al.*, 2006; Feely *et al.*, 2011; Kawarai *et al.*, 2016). Ascertaining the pathogenicity of splice donor or acceptor site variants, which may cause exon skipping, is challenging. For these reasons and in the absence of transcriptomic and variant-specific functional data, nonsense, frameshift and splice donor and acceptor site variants residing in NMD-insensitive regions of the transcript have been classed as variants of uncertain significance in this cohort.

Care should be taken when interpreting novel variants within specific domains and using cross-sectional correlations. For example, the difference both in the average age and range of disease onset between patients carrying a variant at the p.Arg94 amino acid position [average age of onset 4.7 years ± 2.5 years (SD)] and those carrying a variant at the adjacent p.Arg95 amino acid position [average age of onset 31.8 years ± 23.6 years (SD)] is significant. Similarly, patients carrying the p.Arg364Trp variant, often present with an early onset and progressive disease, in contrast to patients carrying the nearby p.Thr362Arg variant which usually presents with symptoms in adulthood and has a more indolent course. Specific *MFN2* variants have been previously described to exhibit interfamilial variability with regards to age of onset, such as the p.Leu741Trp described in two unrelated families, one with average age of onset in the third decade of life and the other with onset of disease in the fifth decade in most members (Dankwa *et al.*, 2018; Lin *et al.*, 2019). Other variants have been reported to show intrafamilial variability such as the p.Arg95Gly with variable clinical severity and significantly different age of onset of symptoms in different affected family members (Dankwa *et al.*, 2019) and p.Leu146Phe with age of onset of disease ranging from childhood to late adulthood for different family members (Klein *et al.*, 2011). A further family in our cohort carrying the dominant p.Ala100Ser variant exhibited intrafamilial heterogeneity, since both siblings had onset of disease in early adulthood and moderate CMTESv2 scores in their forties to fifties, whereas their mother had a late onset neuropathy with a CMTESv2 of 6 at the age of 81.

Our baseline genotype-phenotype correlations, illustrate how CMT2A is an early and severe form of CMT2 with most patients having foot deformities, requiring ankle-foot orthoses and complaining of impaired dexterity at their first visit. At the first visit, the mean CMTESv2 score was 11.06 ± 6.90 (SD) [sample mean age 33.70 ± 19.42 (SD)], the mean CMTNSv2 score was 15.68 ± 9.56 (SD) [sample mean age 36.00 ± 19.78 (SD)] and the mean CMTPedS score was 26.47 ± 10.17 (SD) [sample mean age 10.3 ± 4.36 (SD)], all scores indicative of a moderate burden of disability with CMT2A early in life. A considerable proportion require use of walking aids and are wheelchair-dependent at their first visit and this degree of physical impairment is not encountered in CMT1A (Reilly *et al.*, 2011), CMT1B (Sanmaneechai *et al.*, 2015) or CMTX1 (Panosyan *et al.*, 2017). Interestingly, the majority of patients with CMT2A seem to achieve their gross motor developmental milestones normally as the majority walk at or before the 15th month, yet go on to develop early, and in most cases, severe CMT that progresses faster than other subtypes. This is in contrast to CMT1B, a demyelinating form of CMT, in which the vast majority of patients with an infantile onset show a delay in walking independently, beyond the 15th month (Sanmaneechai *et al.*, 2015). Considering our comparison of baseline characteristics across a range of groups, a childhood onset of disease in AD-CMT2A seems to be the most reliable predictor of significant physical disability accrued and is independent of the disease duration.

The SRM is a popular effect size index used to estimate the responsiveness of outcome measures to clinical change. Based on data from two clinical trials of ascorbic acid in CMT1A (Pareyson *et al.*, 2011; Lewis *et al.*, 2013; Piscosquito *et al.*, 2015), the 1-year SRM of the CMTESv1 was 0.17 indicating minimal responsiveness. Natural history data from a large multicentre CMT1A cohort, also showed a minimally responsive CMTESv2 and CMTESv2-R over 2 years with SRMs of 0.11 and 0.17, respectively (Fridman *et al.*, 2020). By comparison, CMT2A is a more rapidly progressive disease and this is reflected in a 1-year SRM of 0.35 for the CMTESv2 when used in AD-CMT2A. This means that a hypothetical CMT2A double blinded, randomized placebo-controlled trial, powered to detect a complete cessation in disease progression as measured by the CMTESv2 over a 12-month period with 80% power at *P *<* *0.05 significance, would require 131 individuals in each arm. For a treatment trial with a duration of 24 months and using a 2-year SRM of 0.55, the number of individuals needed in each arm would be 53. Complementing clinical assessment tools, biomarkers such as MRI-quantified intramuscular fat accumulation at calf-level are showing promise as a sensitive outcome measure with two studies showing a highly responsive 1-year SRM of 0.83 and 1.04 in CMT1A (Morrow *et al.*, 2016, 2018). Given that CMT2A is a more progressive disease, it is probable that MRI-quantified intramuscular fat accumulation in CMT2A will prove to be an even more sensitive outcome measure in CMT2A and this is currently being investigated. Surprisingly, the Rasch-weighted CMTESv2-R was not sensitive to change at 1 year and had a lower SRM compared to CMTESv2 at a 2-year follow-up. This perceived insensitivity of the CMTESv2-R may have arisen because the psychometrics of the Rasch weighting were performed using CMT1A data which is a more slowly progressive disease compared to CMT2A (Sadjadi *et al.*, 2014). Despite a small sample size of our paediatric CMT2A cohort, analysis of the longitudinal CMTPedS data showed a significant mean change over 1 and 2 years, corresponding to a respective moderate (1-year SRM 0.72) and large responsiveness (2-year SRM 1.06) of this clinical outcome measure. Furthermore, some severe paediatric CMT2A cases reach the ceiling of the outcome score by their early teens and the subsequent plateauing of the clinical scores would give a false impression of disease stabilization. Ultimately, this may make the overall rate of progression seem smaller than it actually is and therefore, a larger paediatric CMT2A cohort is needed to delineate more accurately the progression of CMT2A in childhood.

With the use of next-generation sequencing panels now commonplace, more patients with CMT receive a genetic result than ever before. This has also led to the identification of large numbers of novel variants in *MFN2*, the significance of which are unknown. Large CMT2A cohort studies such as ours are valuable to help investigators curate variants. Moreover, with genetic therapies in development and clinical trials on the horizon, we need to have responsive clinical outcome measures in order to be trial-ready. This study provides evidence that CMTESv2 is a responsive outcome measure for a 2-year clinical trial that, together with the concurrent development of responsive biomarkers, means we are in a good position to perform clinical trials as candidate therapies become available for CMT2A.

## Supplementary Material

awaa323_Supplementary_Data

## Appendix 1

### Additional Inherited Neuropathy Consortium (INC) working group members

Silvia Baratta, Paula Bray, Daniela Calabrese, Kayla Cornett, Gabrielle Donlevy, Katy Eichinger, Maria Foscan, Silvia Genitrini, Natalie Rose Grant, Tara Jones, Diana Lee, Brett A. McCray, Stefania Magri, Manoj Menezes, Krista Mullen, Tina Nanji, Sara Nuzzo, Emanuela Pagliano, Roy Poh, Eun Park, Saba Sadaf, Paola Saveri, Carly Siskind, Janet E. Sowden, Charlotte J. Sumner, Simone Thomas.

## Abbreviations

AD/AR-CMT2A =: autosomal dominant/autosomal recessive Charcot-Marie-Tooth disease type 2A;
CMTESv2(-R) =: Charcot-Marie-Tooth Examination Score v2.0 (Rasch-modified);
CMTNSv2(-R) =: Charcot-Marie-Tooth Neuropathy Score v2.0 (Rasch-modified);
CMTPedS =: Charcot-Marie-Tooth disease Pediatric Scale; SRM = standardized response mean
