# Supplementary material for: Natural history of Charcot-Marie-Tooth disease type 2A: a large international multicentre study
Source: Brain. 2021 Jan 8;143(12):3589–602. doi: 10.1093/brain/awaa323 (PMC7805791; doi:10.1093/brain/awaa323)
Supplement: awaa323_Supplementary_Data [file awaa323_supplementary_data.zip › awaa323-suppl_data/brain-2020-01188-File005.pdf]

Supplementary table 1. The domains of the MFN2 protein and their corresponding topology (conserved domains are in **bold**). Source: Uniprot, O95140 (MFN2_HUMAN).

Domain	Amino acid position(s)	Topology and description
Amino-terminal	1 – 92	Cytosolic
**Dynamin-GTPase**	**93 – 342**	**Cytosolic; GTP-binding domain (nucleotide binding in positions 106-111 and 258-261)**
Spacer	343 – 390	Cytosolic
**Coiled-coil heptad repeat 1 (HR1)**	**391 – 434**	**Cytosolic; helical**
Spacer	435 – 604	Cytosolic
**Transmembrane domain 1 (TM1)**	**605 – 625**	**Outer mitochondrial membrane; helical**
Spacer	626	Mitochondrial intermembrane
**Transmembrane domain 2 (TM2)**	**627 – 647**	**Outer mitochondrial membrane; helical**
Spacer	648 – 694	Cytosolic
**Coiled-coil heptad repeat 2 (HR2)**	**695 – 738**	**Cytosolic; helical**
Carboxyl-terminal	739 – 757	Cytosolic
