# Supplementary material for: Natural history of Charcot-Marie-Tooth disease type 2A: a large international multicentre study
Source: Brain. 2021 Jan 8;143(12):3589–602. doi: 10.1093/brain/awaa323 (PMC7805791; doi:10.1093/brain/awaa323)
Supplement: awaa323_Supplementary_Data [file awaa323_supplementary_data.zip › awaa323-suppl_data/brain-2020-01188-File006.pdf]

Supplementary table 2. Detailed ACMG/AMP pathogenicity criteria used in this study to curate and classify *MFN2* variants accompanied by brief explanations. Abbreviations: AC, allele count; ACMG/AMP, American College of Medical Genetics and Genomics and Association for Molecular Pathology; gnomAD, genome aggregate database; NMD, nonsense mediated decay.

Criterion category	Criterion weight	Criterion	Explanation	References
Pathogenic	Strong	PS4_strong	Total of five or more unrelated probands/families with the same variant and a similar CMT2A phenotype	
		PS3	Evidence from independent well-designed functional studies at a variant level, showing a deleterious effect in mitochondrial morphology and dynamics	Baloh *et al.*, 2007; Detmer and Chan, 2007; Misko *et al.*, 2010
		PP1_strong	Upgraded and used with the appropriate segregation data	Jarvik and Browning, 2016
	Moderate	PM1	Variants in a domain of the *MFN2* gene (amino acid residues 1-390) which shows a statistically significant missense constraint metric; also applied to p.Tyr752X to reflect nonsense variant in last exon escaping NMD	Firth *et al.*, 2009; Havrilla *et al.*, 2019
		PM2	Variant is absent from the gnomAD population database; also applies for heterozygous variants with an AC $\leq$ 3 in gnomAD which would be consistent with the disease prevalence, penetrance, genetic and allelic heterogeneity of AD-CMT2A	Pipis *et al.*, 2019; Ellard *et al.*, 2020
		PM5	Missense variant is at an amino acid residue where a different pathogenic change has been seen before	
		PM6 [a]	Overall points for *de-novo* occurrences: 1	Ellard *et al.*, 2020; ClinGen Recommendation for de novo criteria[a]
		PS4_moderate	Downgraded from strong and used if the variant is observed in a total of 3 or more unrelated probands/families with the same variant and a similar CMT2A phenotype	Ellard *et al.*, 2020

Supplementary table 2. (continued)

Criterion category	Criterion weight	Criterion	Explanation	References
Pathogenic	Moderate	PP1_moderate	Upgraded and used with the appropriate segregation data	Jarvik and Browning, 2016
	Supporting	PP1	Used with the appropriate segregation data	Jarvik and Browning, 2016
		PP3	Missense variant scoring ≥ 0.7 in the REVEL meta-predictor tool	Ioannidis *et al.*, 2016; Ellard *et al.*, 2020
		PS4_supporting	Downgraded from strong and used if the variant is observed in total of 2 unrelated probands/families with the same variant and a similar CMT2A phenotype	Ellard *et al.*, 2020
		PM4_supporting	In-frame deletion or insertion of single amino acid	Ellard *et al.*, 2020
		PM5_supporting	Downgraded from moderate and used if the missense variant is at an amino acid residue where a different *likely* pathogenic change has been seen before	Ellard *et al.*, 2020
		PM6_supporting [a]	Overall points for *de-novo* occurrences: 0.5	Ellard *et al.*, 2020; ClinGen Recommendation for de novo criteria[a]
Benign	Strong	BS1	Heterozygous variants with an AC ≥ 4 in gnomAD would not be consistent with the disease prevalence, penetrance, genetic and allelic heterogeneity of AD-CMT2A	Pipis *et al.*, 2019
		BS2	Published evidence of a variant being observed in a well-documented healthy adult individual was also considered strong evidence for a benign interpretation	

Supplementary table 2. (continued)

Criterion category	Criterion weight	Criterion	Explanation	References
Benign	Strong	BS4	Evidence from pedigrees of non-segregation with disease	
	Supporting	BP4	Missense variant scoring <0.7 in the REVEL meta-predictor tool, suggesting no impact on gene or gene product	Ioannidis *et al.*, 2016; Ellard *et al.*, 2020
		BP5	Found in case with an alternate cause	
		BP7	A synonymous variant not predicted by *in-silico* tools to impact splicing	

[a] *De-novo* variants identified in the current study and previously published cohorts that are cited, were scored with the points-based system that has been developed by the ClinGen Sequence Variant Interpretation group (https://www.clinicalgenome.org/site/assets/files/3461/svi_proposal_for_de_novo_criteria_v1_0.pdf). Given the genetic heterogeneity of CMT2, we only allowed a maximum value of 1 to contribute to the overall score of this criterion and hence this was only used at a moderate (PM6) or supporting level of evidence (PM6_supporting).

Supplementary table 3. Characteristics and frequency of variants which have been classed pathogenic (P, ACMG Class 5) or likely pathogenic (LP, Class 4) in this multicentre CMT2A cohort. Abbreviations: ACMG, American College of Medical Genetics and Genomics; ACMG criteria (in brackets): see main manuscript text and Supplementary Table 2 for explanations; d, cited publication includes a de novo occurrence of the variant; f, cited publication includes family/ies with >1 affected member which can be used for segregation criterion; LP, likely pathogenic; P, pathogenic.

**Variant characteristics**			**Patients (n)**	**Families (n)**	**Pathogenicity classification (ACMG criteria)**	**References**
**Nucleotide substitution**	**Amino acid substitution**	**Inheritance pattern**				
c.227T>C	p.Leu76Pro	Dominant	2	2	LP (PS3, PM2, PS4_moderate, PP3)	This study; Züchner *et al.*, 2004; Verhoeven *et al.*, 2006; Baloh *et al.*, 2007
c.275T>G	p.Leu92Arg	Dominant	1	1	LP (PM1, PM2, PM5, PM6_supporting, PS4_supporting, PP3)	This study; McCorquodale *et al.*, 2011 (d); Chung *et al.*, 2006, Verhoeven *et al.*, 2006 and Choi *et al.*, 2015 published a different amino acid substitute at the same residue
c.280C>T	p.Arg94Trp	Dominant	18	16	P (PS4_strong, PP1_strong, PM1, PM2, PM6, PP3)	This study; Züchner *et al.*, 2004, 2006 (f); Verhoeven *et al.*, 2006 (d, d); Chung *et al.*, 2006 (f); Cho *et al.*, 2007; Detmer and Chan, 2007; Calvo *et al.*, 2009; Braathen *et al.*, 2010 (f); Casasnovas *et al.*, 2010; Nicolaou *et al.*, 2010; Feely *et al.*, 2011; Bombelli *et al.*, 2014; Choi *et al.*, 2015
c.280C>G	p.Arg94Gly	Dominant	2	2	LP (PM1, PM2, PM5, PS4_moderate, PP3)	This study; Feely *et al.*, 2011 published one of these 2 families and has additional 2 families
c.281G>T	p.Arg94Leu	Dominant	1	1	LP (PM1, PM2, PM5, PP3)	This study
c.281G>A	p.Arg94Gln	Dominant	10	9	P (PS3, PS4_strong, PP1_strong, PM6, PP3)	Züchner *et al.*, 2004; Kijima *et al.*, 2005; Verhoeven *et al.*, 2006 (d); Baloh *et al.*, 2007; Neusch *et al.*, 2007 (d); Detmer and Chan, 2007; Banchs *et al.*, 2008 (f); Braathen *et al.*, 2010; Casasnovas *et al.*, 2010 (f); Misko *et al.*, 2010; Feely *et al.*, 2011; Bombelli *et al.*, 2014; Klein *et al.*, 2014 (f)
c.283A>G	p.Arg95Gly	Dominant	8	3	LP (PP1_strong, PM1, PM2, PP3)	This study; Dankwa *et al.*, 2019 published one of these 3 families (f)

Supplementary table 3 (continued).

Variant characteristics			Patients (n)	Families (n)	Pathogenicity classification (ACMG criteria)	References
Nucleotide substitution	Amino acid substitution	Inheritance pattern				
c.292A>G	p.Lys98Glu	Dominant	1	1	LP (PM1, PM2, PS4_supporting, PM5_supporting, PP3)	This study; Vallat *et al.*, 2008; Choi *et al.*, 2015 published a different amino acid substitute at the same residue
c.298G>T	p.Ala100Ser	Dominant	3	1	LP (PM1, PM2, PM5_supporting, PP3)	This study; Verhoeven *et al.*, 2006, Sitarz *et al.*, 2012 and Luigetti *et al.*, 2016 published a different amino acid substitute at the same residue
c.310C>T	p.Arg104Trp	Dominant	9	9	P (PS4_strong, PM1, PM2, PM6, PP1, PP3)	This study; Brockmann *et al.*, 2008 (d); Del Bo *et al.*, 2008 (f, d); Vallat *et al.*, 2008; Calvo *et al.*, 2009 (d); Abe *et al.*, 2011; Baets *et al.*, 2011 (d); Genari *et al.*, 2011; Choi *et al.*, 2015 (d); Tufano *et al.*, 2015; Hoebeke *et al.*, 2018
c.311G>A	p.Arg104Gln	Dominant	1	1	LP (PM1, PM2, PM5, PP3)	This study
c.311G>T	p.Arg104Leu	Dominant	3	2	LP (PM1, PM2, PM5, PS4_supporting, PP3)	This study; Sitarz *et al.*, 2012 included one of these two families
c.313A>G	p.Thr105Ala	Dominant	1	1	LP (PM1, PM2, PM5, PP3)	Sitarz *et al.*, 2012 published this family; Züchner *et al.*, 2004, Lawson *et al.*, 2005, Chung *et al.*, 2006, Feely *et al.*, 2011, Bombelli *et al.*, 2014 and Choi *et al.*, 2015 published a different amino acid substitute at the same residue
c.314C>T	p.Thr105Met	Dominant	1	1	P (PS4_strong, PM1, PM2, PM6, PP3)	Feely *et al.*, 2011 published this family; Züchner *et al.*, 2004; Lawson *et al.*, 2005 (d); Chung *et al.*, 2006 (d); Detmer and Chan, 2007; Bombelli *et al.*, 2014; Choi *et al.*, 2015 (d)
c.326A>G	p.Lys109Arg	Dominant	1	1	LP (PM1, PM2, PM6_supporting, PS4_supporting, PP3)	This study; Ando *et al.*, 2017 (d)
c.392A>C	p.Asn131Thr	Dominant	1	1	LP (PM1, PM2, PM6_Supporting, PP3)	This study (d)

Supplementary table 3 (continued).

Variant characteristics			Patients (n)	Families (n)	Pathogenicity classification (ACMG criteria)	References
Nucleotide substitution	Amino acid substitution	Inheritance pattern				
c.436C>T	p.Leu146Phe	Dominant	1	1	P (PP1_strong, PM1, PM2, PS4_supporting, PP3)	This study; Klein *et al.*, 2011 (f); Bombelli *et al.*, 2014
c.494A>T	p.His165Leu	Dominant	4	1	LP (PM1, PM2, PM5_supporting, PP1, PP3)	Marchesi *et al.*, 2011 published this family; Zhu *et al.*, 2005, Chung *et al.*, 2006, Verhoeven *et al.*, 2006, Cho *et al.*, 2007 and Choi *et al.*, 2015 published a different amino acid substitute at the same residue
c.497C>T	p.Ala166Val	Dominant	2	2	LP (PM1, PM2, PS4_moderate, PM5_supporting, PP3)	This study; Sun *et al.*, 2017 (f); Loiseau *et al.*, 2007 published a different amino acid substitute at the same residue
c.617C>T	p.Thr206Ile	Dominant	1	1	LP (PM1, PM2, PM6 PS4_moderate, PP3)	This study; Verhoeven *et al.*, 2006 (d); Züchner *et al.*, 2006 (d); Choi *et al.*, 2015
c.651T>G	p.Cys217Trp	Dominant	1	1	LP (PM1, PM2, PM5_supporting, PP3)	This study; Xie *et al.*, 2016 published a different amino acid substitute at the same residue
c.695C>A	p.Thr232Asn	Dominant	2	1	LP (PM1, PM2, PM5_supporting, PP3)	Published in Sitarz *et al.*, 2012; Sole *et al.*, 2009 published a different amino acid substitute at the same residue
c.718T>C	p.Phe240Leu	Dominant	1	1	LP (PM1, PM2, PM5_supporting, PP3)	This study; Amiott *et al.*, 2008 published extensive functional studies on a different amino acid substitute at the same residue
c.721T>C	p.Phe241Leu	Dominant	2	1	LP (PM1, PM2, PP1, PP3)	This study (unpublished data: variant segregates with phenotype in 4 affected family members); gnomAD AC: 1, Pipis *et al.*, 2019
c.730G>A	p.Val244Met	Dominant	1	1	P (PS4_strong, PM1, PM2, PM6, PP3)	This study; Kijima *et al.*, 2005; Calvo *et al.*, 2009; Gonzaga-Jauregui *et al.*, 2015 (d, d)
c.742C>G	p.Leu248Val	Dominant	5	2	LP (PM1, PM2, PP1_moderate, PP3)	This study; Feely *et al.*, 2011 published one of these two families

Supplementary table 3 (continued).

Variant characteristics			Patients (n)	Families (n)	Pathogenicity classification (ACMG criteria)	References
Nucleotide substitution	Amino acid substitution	Inheritance pattern				
c.743T>A	p.Leu248His	Dominant	1	1	LP (PM1, PM2, PM5_supporting, PP3)	This study; Feely *et al.*, 2011 published a different amino acid substitute at the same residue
c.745T>A	p.Ser249Thr	Dominant	1	1	LP (PM1, PM2, PM5_supporting, PP3)	This study; Abe *et al.*, 2011 and Xie *et al.*, 2016 published a different amino acid substitute at the same residue
c.746C>G	p.Ser249Cys	Dominant	2	1	LP (PM1, PM2, PM5_supporting, PP3)	This study; Abe *et al.*, 2011 and Xie *et al.*, 2016 published a different amino acid substitute at the same residue
c.751C>T	p.Pro251Ser	Dominant	1	1	LP (PM1, PM2, PS4_supporting, PM5, PP3)	This study; Beaudonnet *et al.*, 2015; Züchner *et al.*, 2004, Feely *et al.*, 2011 and McCorquodale *et al.*, 2011 published a different amino acid substitute at the same residue
c.751C>G	p.Pro251Ala	Dominant	2	1	P (PS3, PM1, PM2, PS4_supporting, PP3)	This study; Züchner *et al.*, 2004; Baloh *et al.*, 2007; Detmer and Chan, 2007
c.752C>G	p.Pro251Arg	Dominant	2	1	LP (PM1, PM2, PM5, PP1, PP3)	Feely *et al.*, 2011 published this family (f); McCorquodale *et al.*, 2011 (f); Züchner *et al.*, 2004 published a different amino acid substitute at the same residue
c.775C>T	p.Arg259Cys	Dominant	2	2	P (PS4_strong, PM1, PM2, PM5_supporting, PP3)	This study; Bombelli *et al.*, 2014; Leonardi *et al.*, 2015 (f); Ajroud-Driss *et al.*, 2009 published a different amino acid substitute which occurred de novo at the same residue
c.788C>T	p.Ser263Phe	Dominant	2	1	LP (PM1, PM2, PM5_supporting, PP3)	This study; Chung *et al.*, 2006, Cho *et al.*, 2007 and Choi *et al.*, 2015 published a different amino acid substitute at the same residue
c.839G>A	p.Arg280His	Dominant	9	8	P (PS3, PS4_strong, PM1, PM6_supporting, PP1, PP3)	This study; Züchner *et al.*, 2004; Chung *et al.*, 2006; Verhoeven *et al.*, 2006 (f); Baloh *et al.*, 2007; Detmer and Chan, 2007; Bombelli *et al.*, 2014; Choi *et al.*, 2015; Hoebeke *et al.*, 2018 (d)

Supplementary table 3 (continued).

Variant characteristics			Patients (n)	Families (n)	Pathogenicity classification (ACMG criteria)	References
Nucleotide substitution	Amino acid substitution	Inheritance pattern				
c.862G>A	p.Glu288Lys	Dominant	1	1	LP (PM1, PM2, PM5_supporting, PP3)	This study; Bergamin *et al.*, 2014 published a different amino acid substitute at the same residue
c.919A>G	p.Lys307Glu	Dominant	1	1	LP (PM1, PM2, PS4_supporting, PP3)	This study; Xie *et al.*, 2016
c.1071G>C	p.Lys357Asn	Dominant	1	1	LP (PM1, PM2, PS4_supporting, PM6_supporting, PP3)	This study; Kijima *et al.*, 2005 (d)
c.1081C>T	p.His361Tyr	Dominant	2	2	P (PS4_strong, PM1, PM2, PM6, PP3)	This study; Verhoeven *et al.*, 2006 (d); Züchner *et al.*, 2006 (d); Misko *et al.*, 2010; Feely *et al.*, 2011
c.1085C>G	p.Thr362Arg	Semi-dominant	5	4	LP (PM1, PM2, PS4_moderate, PP3)	This study
c.1090C>T	p.Arg364Trp	Dominant	15	10	P (PS4_strong, PP1_strong, PM1, PM2, PM6, PP3)	This study; Chung *et al.*, 2006 (d, d); Züchner *et al.*, 2006; Feely *et al.*, 2011 (f); Lin *et al.*, 2011 (d, d); Choi *et al.*, 2012, 2015; Bombelli *et al.*, 2014
c.1091G>A	p.Arg364Gln	Dominant	1	1	LP (PM1, PM2, PM5, PS4_supporting, PP1, PP3)	This study; Loiseau *et al.*, 2007 (f); Banchs *et al.*, 2008 and Casasnovas *et al.*, 2010 describe the same family (f); Chung *et al.*, 2006, Züchner *et al.*, 2006, Calvo *et al.*, 2009, Feely *et al.*, 2011, Lin *et al.*, 2011, Choi *et al.*, 2012, 2015 and Bombelli *et al.*, 2014 published a different amino acid substitute at the same residue
c.1126A>G	p.Met376Val	Dominant	9	6	P (PP1_strong, PM1, PM2, PS4_moderate, PM5_supporting, PP3)	This study; Casasnovas *et al.*, 2010 (f); Vielhaber *et al.*, 2013; Bombelli *et al.*, 2014 published a different amino acid substitute at the same residue

Supplementary table 3 (continued).

Variant characteristics			Patients (n)	Families (n)	Pathogenicity classification (ACMG criteria)	References
Nucleotide substitution	Amino acid substitution	Inheritance pattern				
c.1148C>T	p.Ala383Val	Dominant	6	3	P (PP1_strong, PM1, PM2, PS4_supporting, PP3)	This study; Muglia *et al.*, 2007 and Bergamin *et al.*, 2014 published one of these 3 families (f)
c.1157A>C	p.Gln386Pro	Dominant	1	1	LP (PM1, PM2, PS4_supporting, PM6_supporting, PP3)	This study; Verhoeven *et al.*, 2006 (d)
c.2018T>C	p.Leu673Pro	Dominant	2	1	LP (PP1_strong, PM2, PP3)	Sitarz *et al.*, 2012 published this family (unpublished data: variant segregates with phenotype in 5 affected members)
c.2218T>A	p.Trp740Arg	Dominant	1	1	LP (PM2, PM5, PS4_supporting, PP3)	This study; Lv *et al.*, 2013
c.2219G>C	p.Trp740Ser	Dominant	21	16	P (PS4_strong, PS3, PP1_strong, PM2, PP3)	This study; Züchner *et al.*, 2004; Verhoeven *et al.*, 2006; Baloh *et al.*, 2007; Feely *et al.*, 2011 published one of these 16 families; Gonzaga-Jauregui *et al.*, 2015; Wang *et al.*, 2016
c.2222T>G	p.Leu741Trp	Dominant	4	1	LP (PP1_strong, PM2, PP3)	Dankwa *et al.*, 2018 published this family; Lin *et al.*, 2019 (f)
c.2230G>A	p.Glu744Lys	Dominant	3	2	LP (PM2, PS4_moderate, PM5_supporting, PP3)	This study; Choi *et al.*, 2015; Lin *et al.*, 2011 published a different amino acid substitute at the same residue; another unpublished case listed on Inherited Neuropathy Variant Browser (Saghira *et al.*, 2018)
c.2256C>A	p.Tyr752X	Dominant	1	1	LP (PM1, PM2, PP4_moderate)	Feely *et al.*, 2011 published this family

Supplementary table 4. Characteristics and frequency of variants that have been classed as variants of uncertain significance (VUS, ACMG Class 3) in this multicentre CMT2A cohort. Nonsense, splice acceptor and splice donor variants are further discussed in the main manuscript. Abbreviations: AC, allele count; ACMG, American College of Medical Genetics and Genomics; ACMG criteria (in brackets): see main manuscript text and Supplementary Table 2 for explanations; AOO, age of onset; gnomAD, genome aggregate database; VUS, variant of uncertain significance.

Variant characteristics			Patients (n)	Families (n)	Age of symptom onset for patients	Pathogenicity classification (ACMG criteria)	References
Nucleotide substitution	Amino acid substitution	Inheritance pattern					
IVS2-1G>T	Splice site acceptor	Dominant	2	1	11yrs, 11yrs	VUS	This study
c.334G>A	p.Val112Met	Dominant	2	2	3yrs, 52yrs	VUS (BS1, PM1)	gnomAD AC: 4, Pipis *et al.*, 2019
c.475-7_478delCCATCAGACTG	p.Thr159X	Dominant	1	1	13yrs	VUS (PM2)	This study
c.475-2A>G	Splice site acceptor	Dominant	1	1	18yrs	VUS (PM2)	This study
c.557-558insT	p.Lys187X	Dominant	1	1	8yrs	VUS (PM2)	This study
c.597C>G	p.Asp199Glu	Dominant	1	1	30yrs	VUS (PM1, PM2)	This study
c.653T>C	p.Leu218Pro	Dominant	2	1	12yrs, 28yrs	VUS (PM1, PM2, PP3)	This study
c.711A>T	p.Glu237Asp	Dominant	3	1	3yrs, 4yrs, 6yrs	VUS (PM1, PM2, PP3)	This study
c.725A>G	p.His242Arg	Dominant	1	1	13yrs	VUS (BS2, PM1, PM2)	Variant was observed in a sibling at an age after AOO with no clinical signs of a neuropathy and normal EMG study (unpublished data from one INC centre)

Supplementary table 4. (continued)

Variant characteristics			Patients (n)	Families (n)	Age of symptom onset for patients	Pathogenicity classification (ACMG criteria)	References
**Nucleotide substitution**	**Amino acid substitution**	**Inheritance pattern**					
c.748C>T	p.Arg250Trp	Semi-dominant	1	1	2yrs	VUS (BS1, BS2, PM1, PP3)	This study includes 2 unaffected obligate carriers of R250W in their 5-6th decade of life; Piscosquito *et al.*, 2015 reported a late-onset mild neuropathy in a heterozygous carrier; gnomAD AC: 4, Pipis *et al.*, 2019
c.776G>A	p.Arg259His	Dominant	1	1	56yrs	VUS (BS2, PM1, PM2, PM5, PP3)	This study; Nakhro *et al.*, 2013 and Choi *et al.*, 2015 report the same family (includes one carrier in the family who is asymptomatic); Wu *et al.*, 2018 (sensory-predominant neuropathy); Schabhüttl *et al.*, 2014 published two carriers that were normal by history and EMG studies; gnomAD AC: 1, Pipis *et al.*, 2019

Supplementary table 4. (continued)

Variant characteristics			Patients (n)	Families (n)	Age of symptom onset for patients	Pathogenicity classification (ACMG criteria)	References
Nucleotide substitution	Amino acid substitution	Inheritance pattern					
c.797A>G	p.Glu266Gly	Dominant	1	1	11yrs	VUS (PM1, PM2, PP3)	This study
c.828-830dupGCA	p.Gln276dup	Dominant	1	1	47yrs	VUS (PM1, PM2, PM4_supporting)	This study
c.1105G>C	p.Ala369Pro	Dominant	1	1	Unknown	VUS (PM1, PM2, BP4)	This study
c.1136T>C	p.Leu379Pro	Dominant	1	1	2.5yrs	VUS (PM1, PM2, BP4)	Verhoeven *et al.*, 2006 published an amino acid deletion at the same residue
c.1140-1142CATdel	p.His380_Met381delinsGln	Dominant	2	1	2yrs, 5yrs	VUS (PM1, PM2, PM4_supporting)	This study
c.1160+1G>A	Splice site donor	Dominant	1	1	48yrs	VUS	This study
c.1894C>T	p.Arg632Trp	Dominant	1	1	7yrs	VUS (BS2, PM2)	Hikiami *et al.*, 2018 reports a 64yr old asymptomatic heterozygous carrier of this variant; gnomAD AC: 1, Pipis *et al.*, 2019
c.1910C>T	p.Ser637Phe	Dominant	1	1	47yrs	VUS (PM2, PS4_supporting, PP3)	This study; Bombelli *et al.*, 2014

Supplementary table 4. (continued)

Variant characteristics			Patients (n)	Families (n)	Age of symptom onset for patients	Pathogenicity classification (ACMG criteria)	References
**Nucleotide substitution**	**Amino acid substitution**	**Inheritance pattern**					
c.2213C>T	p.Ala738Val	Dominant	1	1	4yrs	VUS (PM2, PS4_moderate, PP3)	This study; Benedetti *et al.*, 2010; Luigetti *et al.*, 2011; Bergamin *et al.*, 2014 describes the variant in two affectd siblings
c.2234T>G	p.Leu745Arg	Dominant	1	1	12yrs	VUS (PM2, PM5_supporting, PP3)	This study; Calvo *et al.*, 2009 published a different amino acid substitute at the same residue
n/a	Partial gene duplication (exons 4-6)	Dominant	2	1	5yrs, 6yrs	VUS	This study

Supplementary table 5. Clinical characteristics and variant details in pedigrees with autosomal recessive CMT2A (AR-CMT2A) from our study. The table also includes citations of previously published cases with the same variants causing AR-CMT2A. Of note, the variant T362M has been reported in the heterozygous state to cause a late-onset, mild disease consistent with semidominant transmission (Chung *et al.*, 2006; Nicholson *et al.*, 2008; Choi *et al.*, 2015). Abbreviations: AOO, age of onset; CMTESv2, CMT Examination Score version 2; CMTPedS, CMT Pediatric Scale; nd, not done; n/a, not applicable; nil, no further notes.

Pedigree	Patients (n); sex; relationship	Variant characteristics			AOO	Clinical progression			Notes	References
		Nucleotide substitution	Amino acid substitution	Phase		Age at assessment	CMTESv2	CMTPedS		
AR1	1; F	c.113-115delAGA + c.1085C>T	p.Lys38del + p.Thr362Met	Trans	3yrs	46yrs	26	n/a	Nil	Case reported in Polke *et al.*, 2011; T362M also reported in Chung *et al.*, 2006, Nicholson *et al.*, 2008, Vallat *et al.*, 2008, Calvo *et al.*, 2009 and Choi *et al.*, 2015
AR2	1; F	c.449G>T	p.Gly150Val + exon 7&8 deletion	Trans	2yrs	6yrs	n/a	12	Nil	This study; exon 7&8del also reported in Carr *et al.*, 2015
AR3	1; M	c.551C>G + c.1101G>C	p.Pro184Arg + p.Gln367His	Trans	6yrs	39yrs	15	n/a	Nil	This study
AR4	2; M, F; siblings	c.572T>C + c.809T>C	p.Leu191Pro + p.Met270Thr	Trans	11yrs, 7.5yrs	30yrs, 36yrs	nd, 6	n/a	Nil	This study

Supplementary table 5. (continued)

Pedigree	Patients (n); sex; relationship	Variant characteristics			AOO	Clinical progression			Notes	References
		Nucleotide substitution	Amino acid substitution	Phase		Age at assessment	CMTESv2	CMTPedS		
AR5	1; F	c.647T>C	p.Phe216Ser + exon 7&8 deletion	Trans	1.5yrs	18yrs	21	n/a	Nil	Case reported in Polke *et al.*, 2011; F216S also reported in Vallat *et al.*, 2008; exon 7&8del also reported in Carr *et al.*, 2015
AR6	1; F	c.748C>T + c.1426C>T	p.Arg250Trp + p.Arg476X	Trans	10yrs	45yrs	6	n/a	Nil	Case reported in Piscosquito *et al.*, 2015; R250W also reported in Verhoeven *et al.*, 2006
AR7	1; F	c.748C>T	p.Arg250Trp	Apparent homozy.	4yrs	28yrs	10	n/a	Parents (5-6th decade) are unaffected obligate carriers	R250W reported in Verhoeven *et al.*, 2006
AR8	2; M, M; siblings	c.749G>A + c.1085C>G	p.Arg250Gln + p.Thr362Arg	Trans	10yrs, 48yrs	53yrs, 59yrs	19, 14	n/a	Mother who is carrier of T362R has a mild neuropathy and is enrolled in this study	Case reported in Tomaselli *et al.*, 2016

Supplementary table 5. (continued)

Pedigree	Patients (n); sex; relationship	Variant characteristics			AOO	Clinical progression			Notes	References
		Nucleotide substitution	Amino acid substitution	Phase		Age at assessment	CMTESv2	CMTPedS		
AR9	3; M, F, M; siblings	c.838C>G + c.1085C>T	p.Arg280Gly + p.Thr362Met	Unknown	7yrs, 4yrs, 4yrs	30yrs, 25yrs, 30yrs	15, 15, nd	n/a	The variants have only been genetically confirmed in 1 of 3 siblings	T362M also reported in Chung *et al.*, 2006, Nicholson *et al.*, 2008, Vallat *et al.*, 2008, Calvo *et al.*, 2009 and Choi *et al.*, 2015
AR10	1; F	c.922G>T + c.1556G>C	p.Glu308X + p.Arg519Pro	Trans	1.5yrs	20yrs	8	n/a	Nil	Case reported in Polke *et al.*, 2011
AR11	1; M	c.647T>C + c.974delG	p.Phe216Ser + p.Gly325AlafsX9	Trans	2.5yrs	9yrs	13	30	Nil	G325AfsX also reported in Geroldi *et al.*, 2017
AR12	1; F	c.1009-1012delGAGT + c.1085C>T	p.Glu337PhefsX9 + p.Thr362Met	Unknown	2yrs	48yrs	23	n/a	Nil	This study; T362M also reported in Chung *et al.*, 2006, Nicholson *et al.*, 2008, Vallat *et al.*, 2008, Calvo *et al.*, 2009 and Choi *et al.*, 2015

Supplementary table 5. (continued)

Pedigree	Patients (n); sex; relationship	Variant characteristics			AOO	Clinical progression			Notes	References
		Nucleotide substitution	Amino acid substitution	Phase		Age at assessment	CMTESv2	CMTPedS		
AR13	1; M	c.1160+5G>C + c.2119C>T	p.? + p.Arg707Trp	Trans	13yrs	42yrs	13	n/a	*In-silico* tools predict that the intronic variant may have an effect on mRNA splicing consistent with it being a recessive mutation; proband also has upper body and limb lipomata	R707W also reported in Nicholson *et al.*, 2008 and Calvo *et al.*, 2009

Supplementary table 6. Characteristics and frequency of variants that have been classed as likely benign (LB, ACMG Class 2) and benign (B, ACMG Class 1) in this multicentre CMT2A cohort. Abbreviations: AC, allele count; ACMG, American College of Medical Genetics and Genomics; ACMG criteria (in brackets): see main manuscript text and Supplementary Table 2 for explanations; B, benign; gnomAD, genome aggregation database; LB, likely benign; MAF, minor allele frequency.

**Variant characteristics**			**Patients (n)**	**Families (n)**	**AC, MAF (in gnomAD)**	**Pathogenicity classification (ACMG criteria)**	**References**
**Nucleotide substitution**	**Amino acid substitution**	**Inheritance pattern**					
c.892G>A	p.Gly298Arg	Dominant	2	2	606, 0.21%	LB (BS1)	Pipis *et al.*, 2019
c.1181G>A	p.Arg394His	Dominant	1	1	17, 0.007%	LB (BS1)	Pipis *et al.*, 2019
c.1403G>A	p.Arg468His	Dominant	4	4	614, 0.22%	LB (BS1, BP5)	This study; Pipis *et al.*, 2019
c.1452G>A	p.Thr484Thr	Dominant	1	1	246, 0.09%	LB (BS1, BP7)	Pipis *et al.*, 2019
c.1987C>T	p.Arg663Cys	Dominant	1	1	48, 0.02%	LB (BS1)	Pipis *et al.*, 2019
c.2113G>A	p.Val705Ile	Dominant	2	2	1942, 0.69%	B (BS1, BS4)	Albulym *et al.*, 2013 presents evidence of non-segregation; Pipis *et al.*, 2019
c.2119C>T	p.Arg707Trp	Dominant	2	2	71, 0.03%	B (BS1, BS2)	Nicholson *et al.*, 2008 reports a 62yr old asymptomatic heterozygous carrier of this variant; Calvo *et al.*, 2009 reports 2 asymptomatic heterozygous carriers of this variant; Pipis *et al.*, 2019
c.2146G>A	p.Ala716Thr	Dominant	1	1	37, 0.013%	LB (BS1, BP5)	This study; Pipis *et al.*, 2019

References

Abe A, Numakura C, Kijima K, Hayashi M, Hashimoto T, Hayasaka K. Molecular diagnosis and clinical onset of Charcot-Marie-Tooth disease in Japan. J Hum Genet 2011; 56: 364–368.

Ajroud-Driss S, Fecto F, Ajroud K, Yang Y, Donkervoort S, Siddique N, et al. A novel de novo MFN2 mutation causing CMT2A with upper motor neuron signs. Neurogenetics 2009; 10: 359–361.

Albulym OM, Zhu D, Reddel S, Kennerson M, Nicholson G. The MFN2 V705I Variant Is Not a Disease-Causing Mutation: A Segregation Analysis in a CMT2 Family . J Neurodegener Dis 2013; 2013: 1–5.

Amiott EA, Lott P, Soto J, Kang PB, McCaffery JM, DiMauro S, et al. Mitochondrial fusion and function in Charcot-Marie-Tooth type 2A patient fibroblasts with mitofusin 2 mutations. Exp Neurol 2008; 211: 115–127.

Ando M, Hashiguchi A, Okamoto Y, Yoshimura A, Hiramatsu Y, Yuan J, et al. Clinical and genetic diversities of Charcot-Marie-Tooth disease with MFN2 mutations in a large case study. J Peripher Nerv Syst 2017; 22: 191–199.

Baets J, Deconinck T, De Vriendt E, Zimoń M, Yperzeele L, Van Hoorenbeeck K, et al. Genetic spectrum of hereditary neuropathies with onset in the first year of life. Brain 2011; 134: 2664–2676.

Baloh RH, Schmidt RE, Pestronk A, Milbrandt J. Altered axonal mitochondrial transport in the pathogenesis of Charcot-Marie-Tooth disease from mitofusin 2 mutations. J Neurosci 2007; 27: 422–430.

Banchs I, Casasnovas C, Montero J, Martínez-Matos JA, Volpini V. Two Spanish families with Charcot-Marie-Tooth type 2A: Clinical, electrophysiological and molecular findings. Neuromuscul Disord 2008; 18: 974–978.

Beaudonnet G, Cauquil C, Théaudin M, Not A, Labeyrie C, Adam C, et al. Five new mutations in Mitofusin 2-related CMT2 neuropathy. J Peripher Nerv Syst 2015; 20: 88–253.

Benedetti S, Previtali SC, Coviello S, Scarlato M, Cerri F, Di Pierri E, et al. Analyzing Histopathological Features of Rare Charcot-Marie-Tooth Neuropathies to Unravel Their Pathogenesis. Arch Neurol 2010; 67: 1498–1505.

Bergamin G, Boaretto F, Briani C, Pegoraro E, Cacciavillani M, Martinuzzi A, et al. Mutation analysis of MFN2, GJB1, MPZ and PMP22 in Italian patients with axonal Charcot-Marie-Tooth disease. NeuroMolecular Med 2014; 16: 540–550.

Del Bo R, Moggio M, Rango M, Bonato S, D'Angelo MG, Ghezzi S, et al. Mutated mitofusin 2 presents with intrafamilial variability and brain mitochondrial dysfunction. Neurology 2008; 71: 1959–1966.

Bombelli F, Stojkovic T, Dubourg O, Echaniz-Laguna A, Tardieu S, Larcher K, et al. Charcot-Marie-Tooth disease type 2A: From typical to rare phenotypic and genotypic features. JAMA Neurol 2014; 71: 1036–1042.

Braathen GJ, Sand JC, Lobato A, Høyer H, Russell MB. MFN2 point mutations occur in 3.4% of Charcot-Marie-Tooth families. An investigation of 232 Norwegian CMT families. BMC Med Genet 2010; 11

Brockmann K, Dreha-Kulaczewski S, Dechent P, Bönnemann C, Helms G, Kyllerman M, et al. Cerebral involvement in axonal Charcot-Marie-Tooth neuropathy caused by mitofusin2 mutations. J Neurol 2008; 255: 1049–1058.

Calvo J, Funalot B, Ouvrier RA, Lazaro L, Toutain A, De Mas P, et al. Genotype-phenotype correlations in Charcot-Marie-Tooth disease type 2 caused by mitofusin 2 mutations. Arch Neurol 2009; 66: 1511–1516.

Carr AS, Polke JM, Wilson J, Pelayo-Negro AL, Laura M, Nanji T, et al. MFN2 deletion of exons 7 and 8: Founder mutation in the UK population. J Peripher Nerv Syst 2015; 20: 67–71.

Casasnovas C, Banchs I, Cassereau J, Gueguen N, Chevrollier A, Martínez-Matos JA, et al. Phenotypic spectrum of MFN2 mutations in the Spanish population. J Med Genet 2010; 47: 249–256.

Cho HJ, Sung DH, Kim BJ, Ki CS. Mitochondrial GTPase mitofusin 2 mutations in Korean patients with Charcot-Marie-Tooth neuropathy type 2. Clin Genet 2007; 71: 267–272.

Choi BO, Koo SK, Park MH, Rhee H, Yang SJ, Choi KG, et al. Exome sequencing is an efficient tool for genetic screening of Charcot-Marie-Tooth disease. Hum Mutat 2012; 33: 1610–1615.

Choi BO, Nakhro K, Park HJ, Hyun YS, Lee JH, Kanwal S, et al. A cohort study of MFN2 mutations and phenotypic spectrums in Charcot-Marie-Tooth disease 2A patients. Clin Genet 2015; 87: 594–598.

Chung KW, Kim SB, Park KD, Choi KG, Lee JH, Eun HW, et al. Early onset severe and late-onset mild Charcot–Marie–Tooth disease with mitofusin 2 (MFN2) mutations. Brain 2006; 129: 2103–2118.

Dankwa L, Richardson J, Motley WW, Scavina M, Courel S, Bardakjian T, et al. A novel MFN2 mutation causes variable clinical severity in a multi-generational CMT2 family. Neuromuscul Disord 2019; 29: 134–137.

Dankwa L, Richardson J, Motley WW, Züchner S, Scherer SS. A mutation in the heptad repeat 2 domain of MFN2 in a large CMT2A family. J Peripher Nerv Syst 2018; 23: 36–39.

Detmer SA, Chan DC. Complementation between mouse Mfn1 and Mfn2 protects mitochondrial fusion defects caused by CMT2A disease mutations. J Cell Biol 2007; 176: 405–414.

Ellard S, Baple EL, Callaway A, Berry I, Forrester N, Turnbull C, et al. ACGS best practice guidelines for variant classification in rare disease 2020 [Internet]. Assoc Clin Genomic Sci 2020Available from: https://www.acgs.uk.com/quality/best-practice-guidelines/#GeneralGuidelines

Feely SME, Laura M, Siskind CE, Sottile S, Davis M, Gibbons VS, et al. MFN2 mutations cause severe phenotypes in most patients with CMT2A. Neurology 2011; 76: 1690–1696.

Firth H V., Richards SM, Bevan AP, Clayton S, Corpas M, Rajan D, et al. DECIPHER: database of chromosomal imbalance and phenotype in humans using Ensembl resources. Am J Hum Genet 2009; 84: 524–533.

Genari AB, Borghetti VHS, Gouvêa SP, Bueno KC, dos Santos PL, dos Santos AC, et al. Characterizing the phenotypic manifestations of MFN2 R104W

mutation in Charcot-Marie-Tooth type 2. Neuromuscul Disord 2011; 21: 428–432.

Geroldi A, Lastella P, Patruno M, Gotta F, Resta N, Devigili G, et al. Two novel cases of compound heterozygous mutations in mitofusin2: Finding out the inheritance. Neuromuscul Disord 2017; 27: 377–381.

Gonzaga-Jauregui C, Harel T, Gambin T, Kousi M, Griffin LB, Francescatto L, et al. Exome Sequence Analysis Suggests that Genetic Burden Contributes to Phenotypic Variability and Complex Neuropathy. Cell Rep 2015; 12: 1169–1183.

Havrilla JM, Pedersen BS, Layer RM, Quinlan AR. A map of constrained coding regions in the human genome. Nat Genet 2019; 51: 88–95.

Hikiami R, Yamashita H, Koita N, Jingami N, Sawamoto N, Furukawa K, et al. Charcot-Marie-Tooth disease type 2A with an autosomal-recessive inheritance: The first report of an adult-onset disease. J Hum Genet 2018; 63: 89–92.

Hoebeke C, Bonello-Palot N, Audic F, Boulay C, Tufod D, Attarian S, et al. Retrospective study of 75 children with peripheral inherited neuropathy: Genotype–phenotype correlations. Arch Pediatr 2018; 25: 452–458.

Ioannidis NM, Rothstein JH, Pejaver V, Middha S, McDonnell SK, Baheti S, et al. REVEL: an Ensemble method for predicting the pathogenicity of rare missense variants. Am J Hum Genet 2016; 99: 877–885.

Jarvik GP, Browning BL. Consideration of cosegregation in the pathogenicity classification of genomic variants. Am J Hum Genet 2016; 98: 1077–1081.

Kijima K, Numakura C, Izumino H, Umetsu K, Nezu A, Shiiki T, et al. Mitochondrial GTPase mitofusin 2 mutation in Charcot-Marie-Tooth neuropathy type 2A. Hum Genet 2005; 116: 23–27.

Klein CJ, Kimmel GW, Pittock SJ, Engelstad JNE, Cunningham JM, Wu Y, et al. Large kindred evaluation of mitofusin 2 novel mutation, extremes of neurologic presentations, and preserved nerve mitochondria. Arch Neurol 2011; 68: 1295–1302.

Klein CJ, Middha S, Duan X, Wu Y, Litchy WJ, Gu W, et al. Application of whole exome sequencing in undiagnosed inherited polyneuropathies. J Neurol Neurosurg Psychiatry 2014; 85: 1265–1272.

Lawson VH, Graham B V., Flanigan KM. Clinical and electrophysiologic features of CMT2A with mutations in the mitofusin 2 gene. Neurology 2005; 65: 197–204.

Leonardi L, Marcotulli C, Storti E, Tessa A, Serrao M, Parisi V, et al. Acute optic neuropathy associated with a novel MFN2 mutation. J Neurol 2015; 262: 1678–1680.

Lin HP, Ho KWD, Jerath NU. Late onset CMT2A in a family with an MFN2 variant: c.2222T>G (p.Leu741Trp). J Neuromuscul Dis 2019; 6: 259–261.

Lin KP, Soong BW, Yang CC, Huang LW, Chang MH, Lee IH, et al. The mutational spectrum in a cohort of Charcot-Marie-Tooth disease type 2 among the han Chinese in Taiwan. PLoS One 2011; 6

Loiseau D, Chevrollier A, Verny C, Guillet V, Gueguen N, De Crescenzo MAP, et al. Mitochondrial coupling defect in Charcot-Marie-Tooth type 2A disease. Ann Neurol 2007; 61: 315–323.

Luigetti M, Fabrizi GM, Bisogni G, Romano A, Taioli F, Ferrarini M, et al. Charcot-Marie-Tooth type 2 and distal hereditary motor neuropathy: Clinical, neurophysiological and genetic findings from a single-centre experience. Clin Neurol Neurosurg 2016; 144: 67–71.

Luigetti M, Fabrizi GM, Taioli F, Conte A, Del Grande A, Sabatelli M. Clinical, electrophysiological and pathological findings of a patient with CMT2 due to the p.Ala738Val mitofusin 2 mutation. J Neurol Sci 2011; 307: 168–170.

Lv H, Wang L, Li W, Qiao X, Li Y, Wang Z, et al. Mitofusin 2 gene mutation causing early-onset CMT2A with different progressive courses. Clin Neuropathol 2013; 32: 16–23.

Marchesi C, Ciano C, Salsano E, Nanetti L, Milani M, Gellera C, et al. Co-occurrence of amyotrophic lateral sclerosis and Charcot-Marie-Tooth disease type 2A in a patient with a novel mutation in the mitofusin-2 gene. Neuromuscul Disord 2011; 21: 129–131.

McCorquodale DS, Montenegro G, Peguero A, Carlson N, Speziani F, Price J, et al. Mutation screening of mitofusin 2 in Charcot-Marie-Tooth disease type 2. J Neurol 2011; 258: 1234–1239.

Misko A, Jiang S, Wegorzewska I, Milbrandt J, Baloh RH. Mitofusin 2 is necessary for transport of axonal mitochondria and interacts with the Miro/Milton complex. J Neurosci 2010; 30: 4232–4240.

Muglia M, Vazza G, Patitucci A, Milani M, Pareyson D, Taroni F, et al. A novel founder mutation in the MFN2 gene associated with variable Charcot-Marie-Tooth type 2 phenotype in two families from Southern Italy [5]. J Neurol Neurosurg Psychiatry 2007; 78: 1286–1287.

Nakhro K, Park JM, Choi BO, Chung KW. Missense mutations of mitofusin 2 in axonal Charcot-Marie-Tooth neuropathy: Polymorphic or incomplete penetration? Animal Cells Syst (Seoul) 2013; 17: 228–236.

Neusch C, Senderek J, Eggermann T, Elolff E, Bähr M, Schneider-Gold C. Mitofusin 2 gene mutation (R94Q) causing severe early-onset axonal polyneuropathy (CMT2A). Eur J Neurol 2007; 14: 575–577.

Nicholson GA, Magdelaine C, Zhu D, Grew S, Ryan MM, Sturtz F, et al. Severe early-onset axonal neuropathy with homozygous and compound heterozygous MFN2 mutations. Neurology 2008; 70: 1678–1681.

Nicolaou P, Zamba-Papanicolaou E, Koutsou P, Kleopa KA, Georghiou A, Hadjigeorgiou G, et al. Charcot-marie-tooth disease in Cyprus: Epidemiological, clinical and genetic characteristics. Neuroepidemiology 2010; 35: 171–177.

Pipis M, Rossor AM, Laura M, Reilly MM. Next-generation sequencing in Charcot-Marie-Tooth disease: opportunities and challenges. Nat Rev Neurol 2019; 15: 644–656.

Piscosquito G, Saveri P, Magri S, Ciano C, Di Bella D, Milani M, et al. Mutational mechanisms in MFN2 -related neuropathy: Compound heterozygosity for recessive and semidominant mutations. J Peripher Nerv Syst 2015; 20: 380–386.

Polke JM, Laurá M, Pareyson D, Taroni F, Milani M, Bergamin G, et al. Recessive axonal Charcot-Marie-Tooth disease due to compound heterozygous mitofusin 2 mutations. Neurology 2011; 77: 168–173.

Saghira C, Bis DM, Stanek D, Strickland A, Herrmann DN, Reilly MM, et al. Variant pathogenicity evaluation in the community-driven Inherited Neuropathy

Variant Browser. Hum Mutat 2018; 39: 635–642.

Schabhüttl M, Wieland T, Senderek J, Baets J, Timmerman V, De Jonghe P, et al. Whole-exome sequencing in patients with inherited neuropathies: Outcome and challenges. J Neurol 2014; 261: 970–982.

Sitarz KS, Yu-Wai-Man P, Pyle A, Stewart JD, Rautenstrauss B, Seeman P, et al. MFN2 mutations cause compensatory mitochondrial DNA proliferation. Brain 2012; 135: 1–3.

Sole G, Ferrer X, Vital C, Martin-Negrier ML, Vital A, Latour P. Ultrastructural mitochondrial modifications characteristic of mitofusin 2 mutations (CMT2A). J Peripher Nerv Syst 2009; 14: 206–207.

Sun B, Chen Z, Ling L, Yang F, Huang X. Clinical and genetic spectra of Charcot-Marie-Tooth disease in Chinese Han patients. J Peripher Nerv Syst 2017; 22: 13–18.

Tomaselli PJ, Rossor AM, Polke JM, Poh R, Blake J, Reilly MM. Semi-dominant mutations in MFN2-related neuropathy and implications for genetic counselling. J Peripher Nerv Syst 2016; 21: 52–54.

Tufano M, Cappuccio G, Terrone G, Manganelli F, Pisciotta C, Geroldi A, et al. Early onset Charcot-Marie-Tooth neuropathy type 2A and severe developmental delay: expanding the clinical phenotype of MFN2-related neuropathy. J Peripher Nerv Syst 2015; 20: 415–418.

Vallat JM, Ouvrier RA, Pollard JD, Magdelaine C, Zhu D, Nicholson GA, et al. Histopathological findings in hereditary motor and sensory neuropathy of axonal type with onset in early childhood associated with mitofusin 2 mutations. J Neuropathol Exp Neurol 2008; 67: 1097–1102.

Verhoeven K, Claeys KG, Züchner S, Schröder JM, Weis J, Ceuterick C, et al. MFN2 mutation distribution and genotype/phenotype correlation in Charcot-Marie-Tooth type 2. Brain 2006; 129: 2093–2102.

Vielhaber S, Grazyna B, Bullet D-V, Peeva V, Schoeler S, Kudin AP, et al. Mitofusin 2 mutations affect mitochondrial function by mitochondrial DNA depletion. Acta Neuropathol 2013; 125: 245–256.

Wang W, Wang C, Brian Dawson D, Thorland EC, Lundquist PA, Eckloff BW, et al. Target-enrichment sequencing and copy number evaluation in inherited polyneuropathy. Neurology 2016; 86: 1762–1771.

Wu R, Fu J, Meng L, Lv H, Wang Z, Yuan Y. Late-onset hereditary sensory and autonomic neuropathy expands the phenotypic spectrum of MFN2-related diseases. Neuropathology 2018; 38: 463–467.

Xie Y, Li X, Liu L, Hu Z, Huang S, Zhan Y, et al. MFN2-related genetic and clinical features in a cohort of Chinese CMT2 patients. J Peripher Nerv Syst 2016; 21: 38–44.

Zhu D, Kennerson M, Walizada G, Zuchner S, Vance J, Nicholson G. Charcot–Marie–Tooth with pyramidal signs is genetically heterogeneous: Families with and without MFN2 mutations. Neurology 2005; 65: 496–497.

Züchner S, De Jonghe P, Jordanova A, Claeys KG, Guergueltcheva V, Cherninkova S, et al. Axonal neuropathy with optic atrophy is caused by mutations in mitofusin 2. Ann Neurol 2006; 59: 276–281.

Züchner S, Mersiyanova I V., Muglia M, Bissar-Tadmouri N, Rochelle J, Dadali EL, et al. Mutations in the mitochondrial GTPase mitofusin 2 cause Charcot-Marie-Tooth neuropathy type 2A. Nat Genet 2004; 36: 449–451.
